# Supplementary material for: Atomic overlayer of permeable microporous cuprous oxide on palladium promotes hydrogenation catalysis
Source: Nat Commun. 2022 May 11;13:2597. doi: 10.1038/s41467-022-30327-2 (PMC9095604; doi:10.1038/s41467-022-30327-2)
Supplement: Supplementary file 1 — Supplementary Information [file 41467_2022_30327_MOESM1_ESM.pdf]

Supplementary Information for

Atomic Overlayer of Permeable Microporous Cuprous Oxide on Palladium Promotes Hydrogenation Catalysis

Kunlong Liu,[1,†] Lizhi Jiang,[1,2,†] Wugen Huang,[3,4,†] Guozhen Zhu,[5] Yue-Jiao Zhang,[1] Chaofa Xu,[1] Ruixuan Qin,[1] Pengxin Liu,[1] Chengyi Hu,[1] Jingjuan Wang,[1] Jian-Feng Li,[1] Fan Yang,[3,6,*] Gang Fu,[1,7,*] and Nanfeng Zheng[1,7,*]

Affiliations:

[1] State Key Laboratory for Physical Chemistry of Solid Surfaces, Collaborative Innovation Center of Chemistry for Energy Materials, and National & Local Joint Engineering Research Center for Preparation Technology of Nanomaterials, College of Chemistry and Chemical Engineering, Xiamen University, Xiamen 361005, China
[2] The Straits Institute of Flexible Electronics (SIFE, Future Technologies), Fujian Normal University, Fuzhou 350117, China
[3] State Key Laboratory of Catalysis, Dalian Institute of Chemical Physics, Chinese Academy of Sciences, Dalian 116023, China
[4] University of Chinese Academy of Sciences, Beijing 100049, China
[5] Department of Mechanical Engineering and Manitoba Institute of Materials, University of Manitoba, Winnipeg, MB, R3T 5V6, Canada
[6] School of Physical Science and Technology, ShanghaiTech University, Shanghai 201210, China
[7] Innovation Laboratory for Sciences and Technologies of Energy Materials of Fujian Province (IKKEM), Xiamen 361102, China
[†] These authors contributed equally: Kunlong Liu, Lizhi Jiang, Wugen Huang
* Corresponding authors.
E-mail: nfzheng@xmu.edu.cn (N. F. Zheng); gfu@xmu.edu.cn (G. Fu); fyang@shanghaitech.edu.cn (F. Yang)

Table of Contents:

Supplementary Methods
Supplementary Figures
Supplementary Tables
Supplementary References

Supplementary Methods

Materials. Palladium (II) acetylacetonate [Pd(acac)$_2$] (99%) was purchased from Kunming Institute of Precious Metals. Poly(vinylpyrrolidone) (PVP, MW=30 000, AR), Tetrabutylammonium bromide (TBAB), Acetone (C_3H_6O), Cupric nitrate [Cu(NO$_3$)$_2$], N,N-dimethylformamide (DMF), Methanol (CH_3OH), and Ethanol (C_2H_5OH) were purchased from Sinopharm Chemical Reagent Co. Ltd. (Shanghai, China). Phenylacetylene (PhC≡CH), Methanol-D4 (CD_3OD), Styrene (STY), and other alkynes were purchased from Aladdin. Commercial Pd/C was purchased from Alfa Aesar. H_2 (99.99%), D_2 (99.999%), CO (99.999%) and N_2 (99.999%) were purchased from Linde Gas Co. Ltd. The water used in all experiment was deionized water (18.25 MΩ). All chemicals were used as received without further purification.

TEM characterizations. For TEM characterizations, the samples were dispersed in ethanol and dropped onto 300-mesh carbon-coated copper (or gold) grids. The solvent was evaporated in air naturally. TEM characterizations were carried out on a TECNAI F30 transmission electron microscope operating at 300 kV.

High-resolution TEM characterizations. High-resolution TEM was performed on a JEOL200F transmission electron microscope operated at 200 keV. Both annular-bright-field (ABF) and high-angle annular-dark-field (HAADF) images were acquired with an illumination semi-angle of 25 mrad and probe current of 100 pA.

Electrochemical measurements. Ethanol dispersion of purified nanosheets was deposited on glassy carbon electrode to obtain the working electrodes after the solvent was dried by an IR lamp. A saturated calomel electrode (SCE) and a platinum foil were used as the reference and counter electrode, respectively. The cyclic voltammograms were recorded at a sweep rate of 50 $mV \cdot s^{-1}$ in 0.5M H_2SO_4. Before cyclic voltammetry (CV) measurements, the electrolyte was purged with high purity N_2 gas for at least 30 min to ensure the gas saturated.

Temperature-programmed desorption mass spectrometry (TPD-MS). The TPD-MS experiment was performed on an in-house-built TPD-TOF analyzer. ~3.0 mg of sample was pyrolyzed in a small ceramic tube with heating coil powered by a precise electric source and adjusted in intervals of 10 mV. A K-type thermocouple was inserted at the other end of ceramic tube insulated the catalyst powder. The temperature of the sample tube was ramped smoothly from room temperature to 600 °C at a rate of 10 °C min^{-1}, controlled by a computer. The desorbed species were ionized by an ultraviolet lamp with a photon energy of 10.6 eV positioned very close to the sample tube. Then the information transferred to the TOF analyzer had a resolution of better than 5,000 and a sensitivity at the ppb level by an ion optical system. All of these steps were processed in a high vacuum (∼5×10^{-5} Pa). The mass spectrum and sample temperature

were acquired and recorded every second. Each spectrum is an accumulation of 10,000 spectra gathered at intervals of 100 μs.

X-ray absorption experiments and analysis. The X-ray absorption spectra (XAS) were recorded at the XAS station (BL14W1) of the Shanghai Synchrotron Radiation Facility (SSRF)[1-3]. The electron storage ring was operated at 3.5 GeV. Si(311) double-crystal was used as the monochromator, and the data was collected using a solid-state detector under ambient conditions (Si(111) for Cu K-edge). The beam size was limited by the horizontal and vertical slits with the area of 1 × 4 mm^2 during XAFS measurements. The X-ray absorption of Pd foil at Pd K-edge (Cu foil at Cu K-edge) was measured for energy calibration and data processing stander. The as-obtained PdCu@Cu_2O with different Cu/Pd ratio were measured under ambient atmosphere. The as-obtained XAFS data were processed in Athena (version 0.9.26) for background, pre-edge line and post-edge line calibration.

Diffuse reflectance FTIR characterizations. *In situ* FTIR was carried out by using 100 mg catalyst that was pressed into a small disc and then transferred into an in situ chamber of FTIR. The chamber was flushed with 1 bar N_2 at room temperature ten times and then recorded by FTIR (Thermo Fisher IS50). After, PhC≡CH was carried into the chamber by N_2 for 20 min. Then, H_2 was introduced and keeps for 80 min. The blank chamber with N_2 was used for background correction.

Nuclear Magnetic Resonance (NMR). The ^{1}H-NMR and ^{2}H-NMR spectra were recorded on an AVANCE III HD 500 MHz spectrometer in CH_3OH or CD_3OD.

Inductively coupled plasma-mass spectrometry (ICP-MS) measurements. The contents of Pd and Cu were measured by an ICP-MS (Agilent 7700x, USA).

X-ray photoelectron spectroscopy. X-ray photoelectron spectroscopy data were acquired using a Kratos Axis Ultra X-ray photoelectron spectroscope incorporated with a 165 mm hemispherical electron energy analyzer. The incident radiation was monochromatic Al Kα X-rays at 150 W. Survey (wide) scans were taken at an analyzer pass energy of 160 eV and multiplex (narrow) high-resolution scans of Cu *2p* at a pass energy of 40 eV. Survey scans were carried out over a 1,200 eV binding energy range with 1.0 eV steps and a dwell time of 100 ms. Narrow high-resolution scans were run with 0.1 eV steps and 162 ms dwell time

High-sensitivity low-energy ion scattering spectroscopy (LEISS) measurements. The HS-LEISS measurements were carried out on an Ion-TOF Qtac100 low energy ion scattering analyzer. Ne^+ ions with a kinetic energy of 5 keV were applied at a low ion flux of 1600 $pA \cdot cm^{-2}$. The scattering angle was 145°.

Thermo-gravimetric analysis (TGA) characterizations. The TGA experiments were conducted on a SDT-Q600 under N_2 atmosphere with a heating rate of 10 K/min.

Supplementary Figures

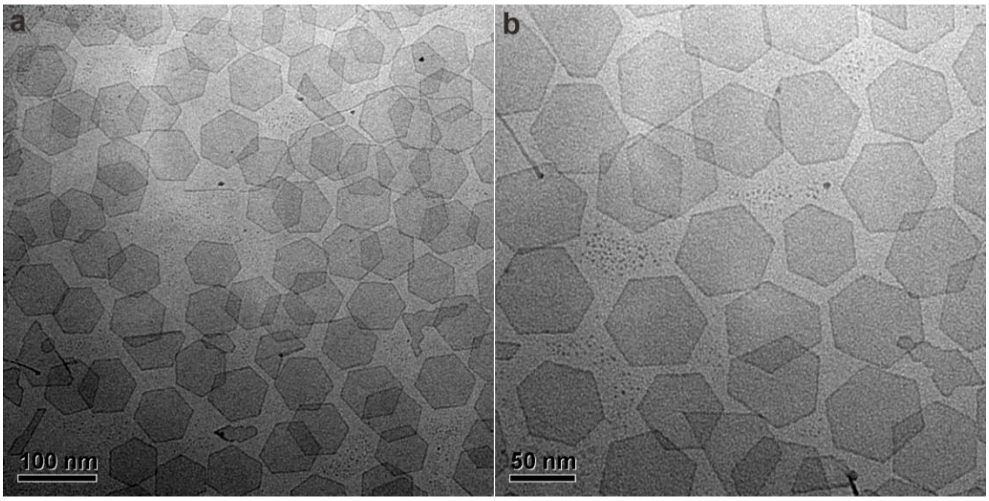

Supplementary Figure 1. Representative TEM images of Pd NSs (a, b).

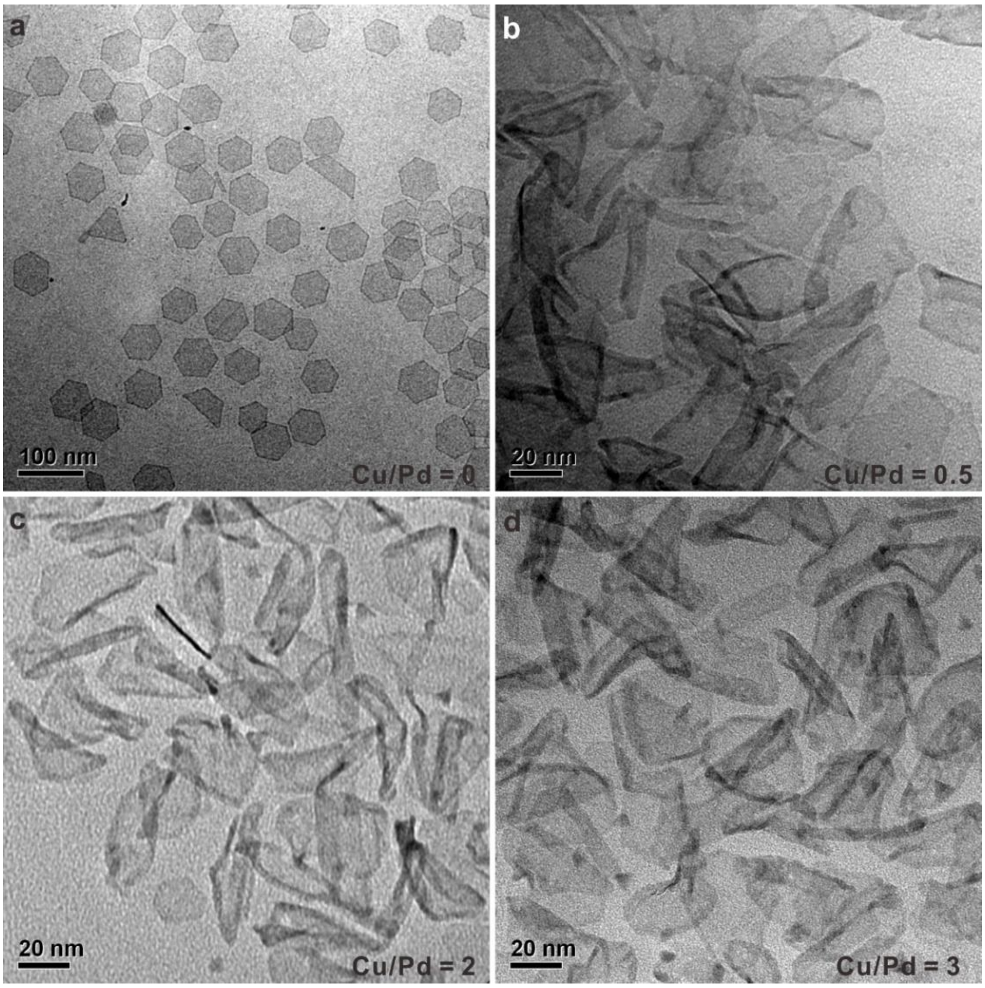

Supplementary Figure 2. Representative TEM images of PdCu@Cu_2O with different Cu/Pd ratio. (a) Cu/Pd = 0. (b) Cu/Pd = 0.5. (c) Cu/Pd = 2. (d) Cu/Pd = 3.

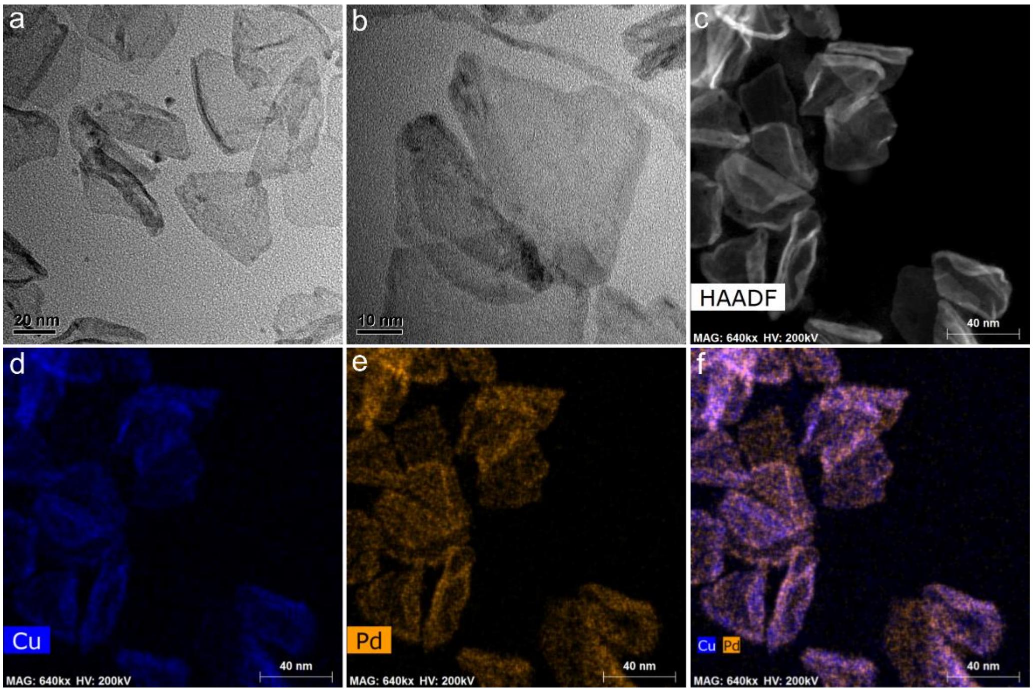

Supplementary Figure 3. TEM characteristic of PdCu@Cu_2O (Cu/Pd = 1). (a, b) Representative HRTEM images. (c) Representative HAADF images. (d-f) EDX mapping images.

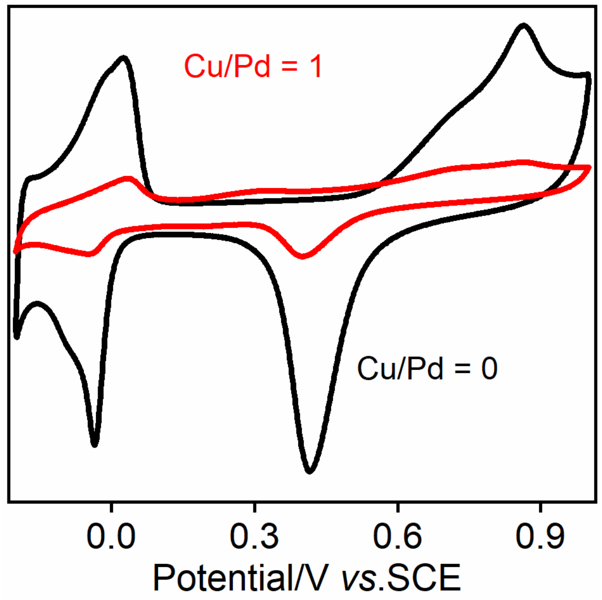

Supplementary Figure 4. Cyclic voltammetry (CV) curves of PdCu@Cu_2O (Cu/Pd = 1) and Pd NSs.

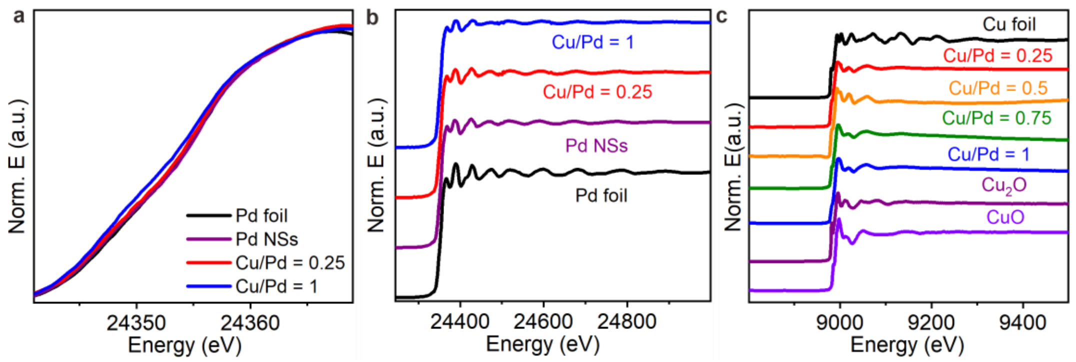

Supplementary Figure 5. X-ray absorption near edge structure spectra (XANES) of (a, b) Pd K-edge and (c) Cu K-edge for PdCu@Cu_2O with different Cu/Pd ratios and references.

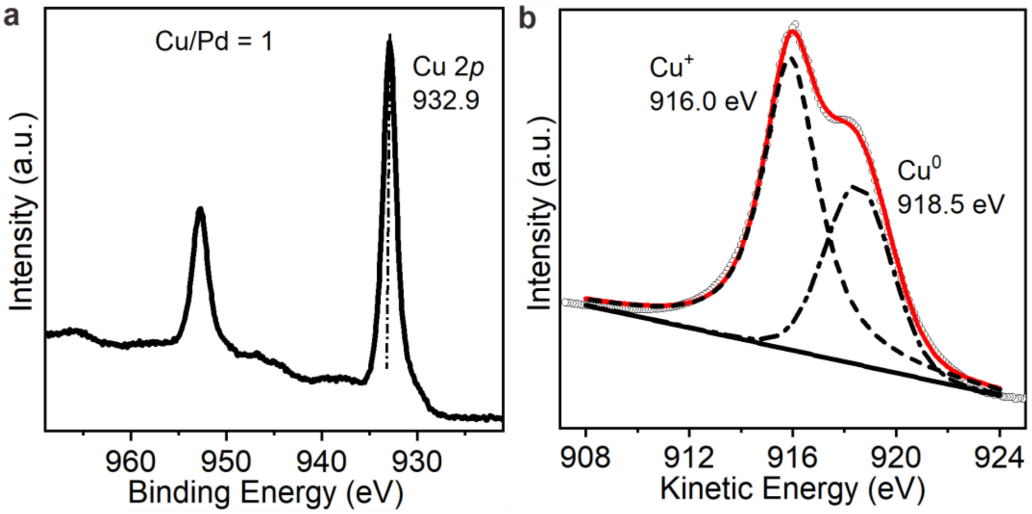

Supplementary Figure 6. The XPS spectra of Cu in PdCu@Cu_2O (Cu/Pd = 1). (a) Cu 2p XPS. (b) Cu LMM XAES spectra.

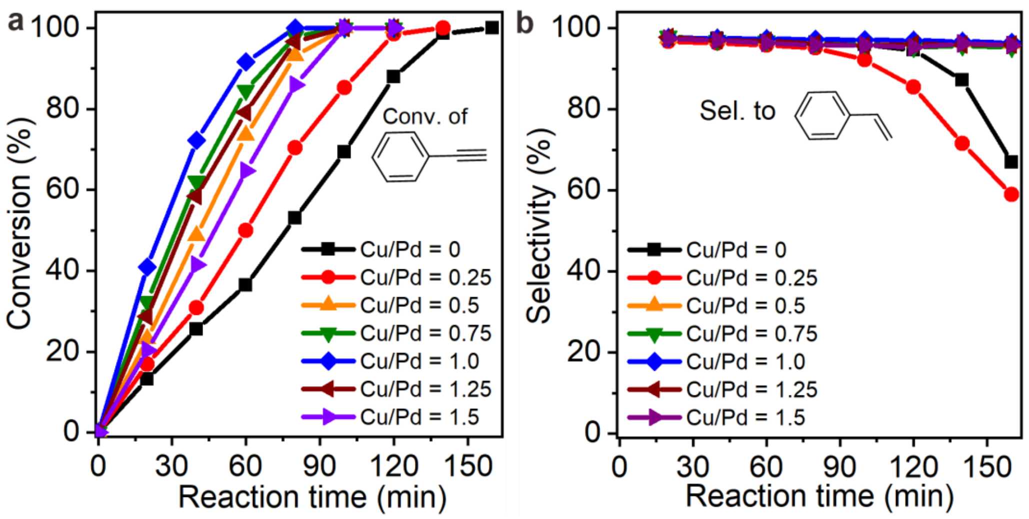

Supplementary Figure 7. Catalytic performance of semi-hydrogenation of PhC≡CH over PdCu@Cu_2O with different Cu/Pd ratio. (a) Time-dependent activity. (b) Time-dependent selectivity. Reaction conditions: 10 mL ethanol; 2 μmol Pd; 4 mmol PhC≡CH (1:2,000); T = 303 K; pressure = 0.1 MPa H_2.

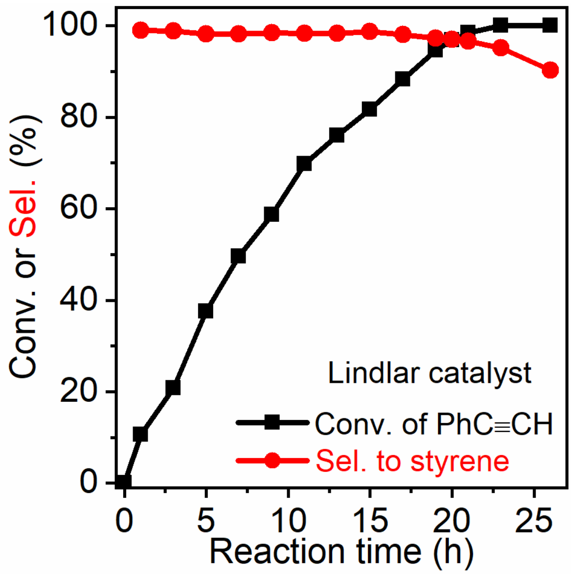

Supplementary Figure 8. Time-dependent catalysis of semi-hydrogenation of PhC≡CH over Lindlar catalyst. Reaction conditions: 10 mL ethanol; 2 μmol Pd; 1 mmol PhC≡CH (1:500); T = 303 K; pressure = 0.1 MPa H_2.

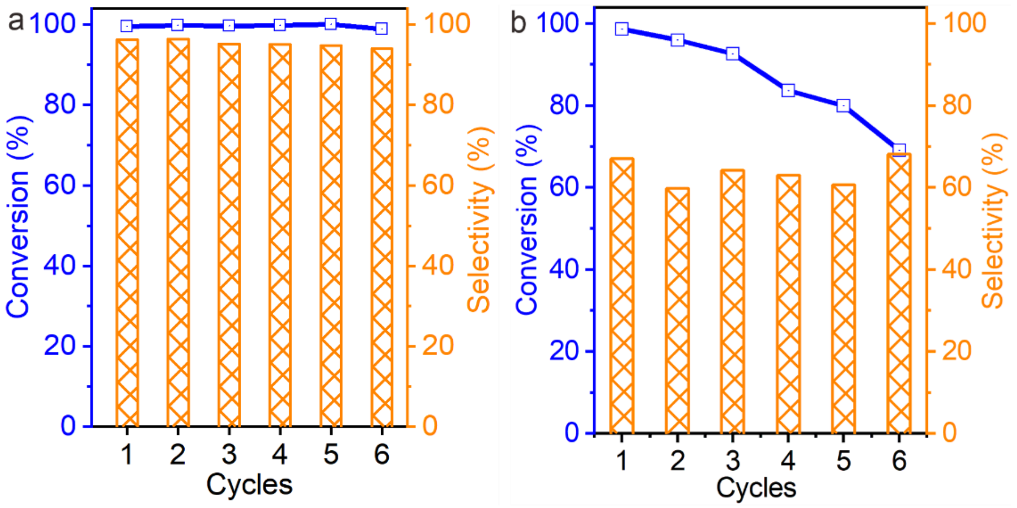

Supplementary Figure 9. Catalytic stability of semi-hydrogenation of PhC≡CH over (a) PdCu@Cu_2O (Cu/Pd = 1) and (b) Pd NSs. Reaction conditions: 10 mL ethanol; 2 µmol Pd; 4 mmol PhC≡CH (1:2,000); T = 303 K; pressure = 0.1 MPa H_2.

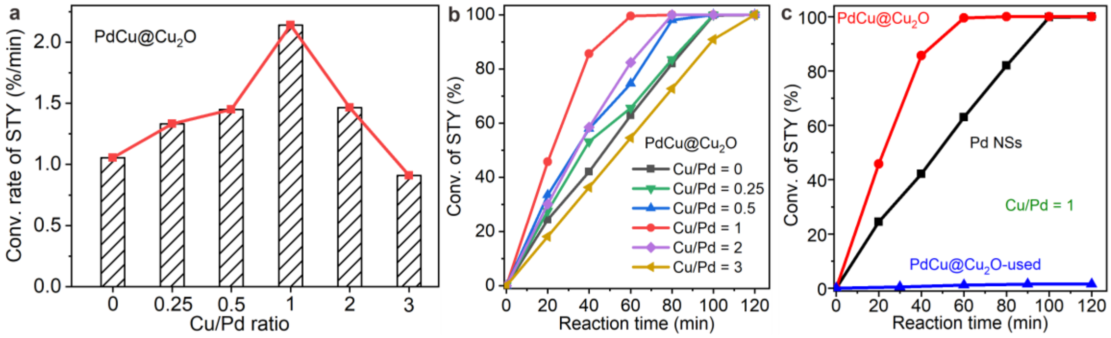

Supplementary Figure 10. Catalytic performance of styrene hydrogenation over PdCu@Cu_2O and references. (a) Conversion rate of styrene hydrogenation. (b) Time-dependent catalysis of styrene hydrogenation catalyzed by PdCu@Cu_2O with different Cu/Pd ratio. (c) Time-dependent catalysis of styrene hydrogenation catalyzed by PdCu@Cu_2O after semi-hydrogenation of PhC≡CH and references. Reaction conditions: 10 mL ethanol; 2 µmol Pd; 4 mmol Styrene (1:2,000); T = 303 K; pressure = 0.1 MPa H_2.

Note: The fresh PdCu@Cu_2O catalyst with the ratio of Cu/Pd ≤ 2 showed higher catalytic activity in styrene hydrogenation than that of Pd NSs under the same reaction conditions.

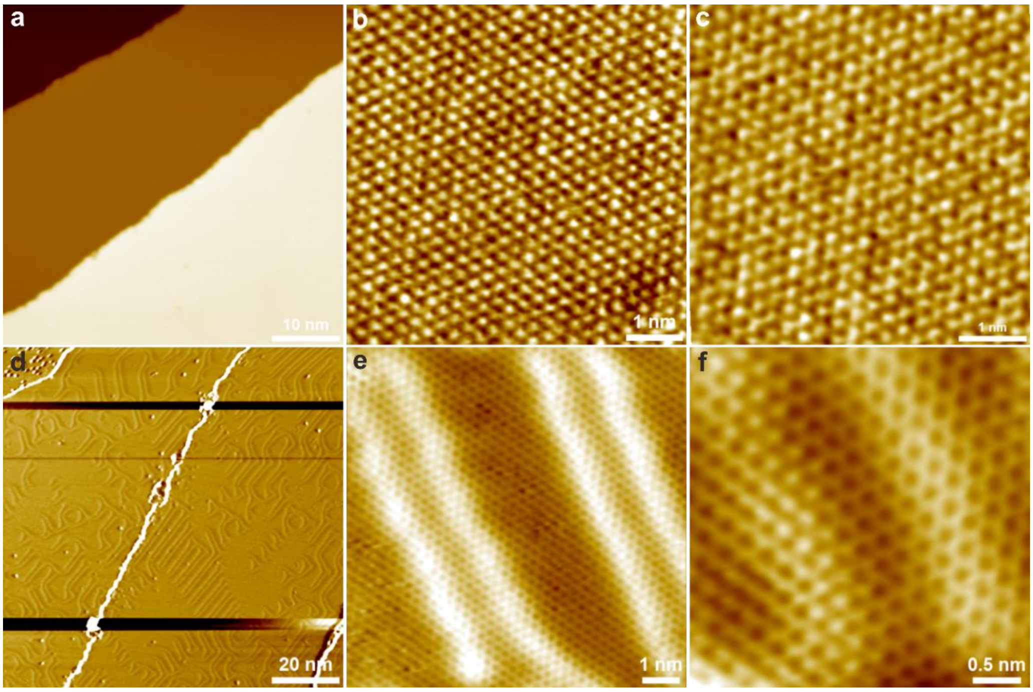

Supplementary Figure 11. Scanning tunneling microscope (STM) images of (a-c) Pd(111) and (d-f) Pd(111) with ~0.96 ML deposited Cu. Scanning parameters: (a) $V_s = 1.1$ V, $I = 0.7$ nA; (b) $V_s = 0.005$ V, $I = 5.1$ nA; (c) $V_s = 0.004$ V, $I = 4.6$ nA; (d) $V_s = 1.0$ V, $I = 0.1$ nA; (e) $V_s = 0.1$ V, $I = 3.0$ nA; (f) $V_s = 0.05$ V, $I = 4.1$ nA.

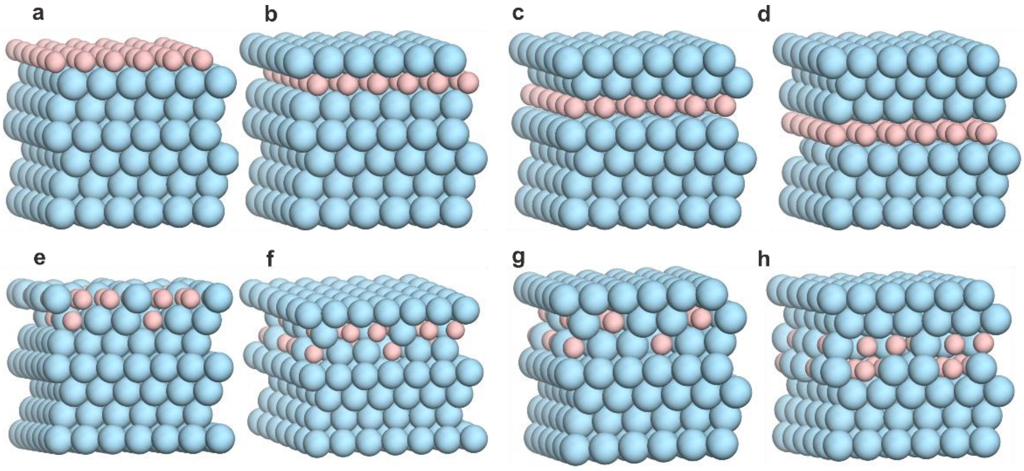

Supplementary Figure 12. Structures of different arrangement of Cu atoms into Pd(111) lattice with the ratio of Cu/Pd = 1/6 (a-h).

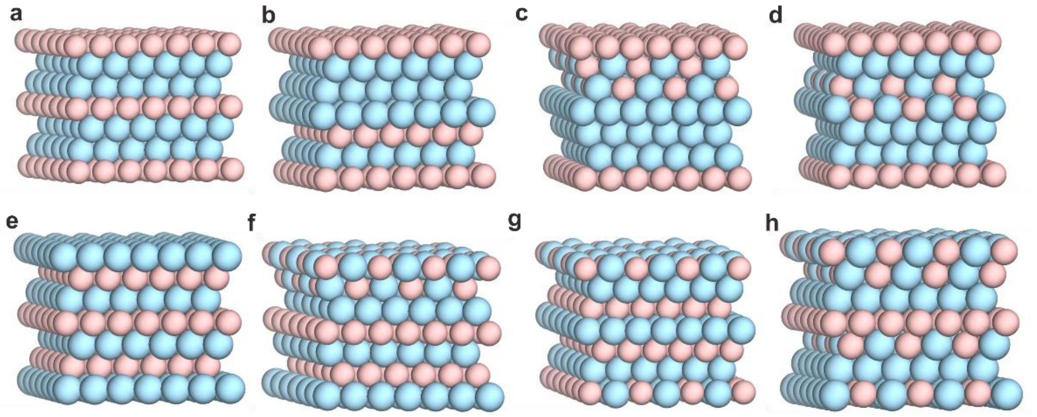

Supplementary Figure 13. Structures of different arrangement of Cu atoms on Pd(111) lattice with the ratio of Cu/Pd = 3/4 (a-h).

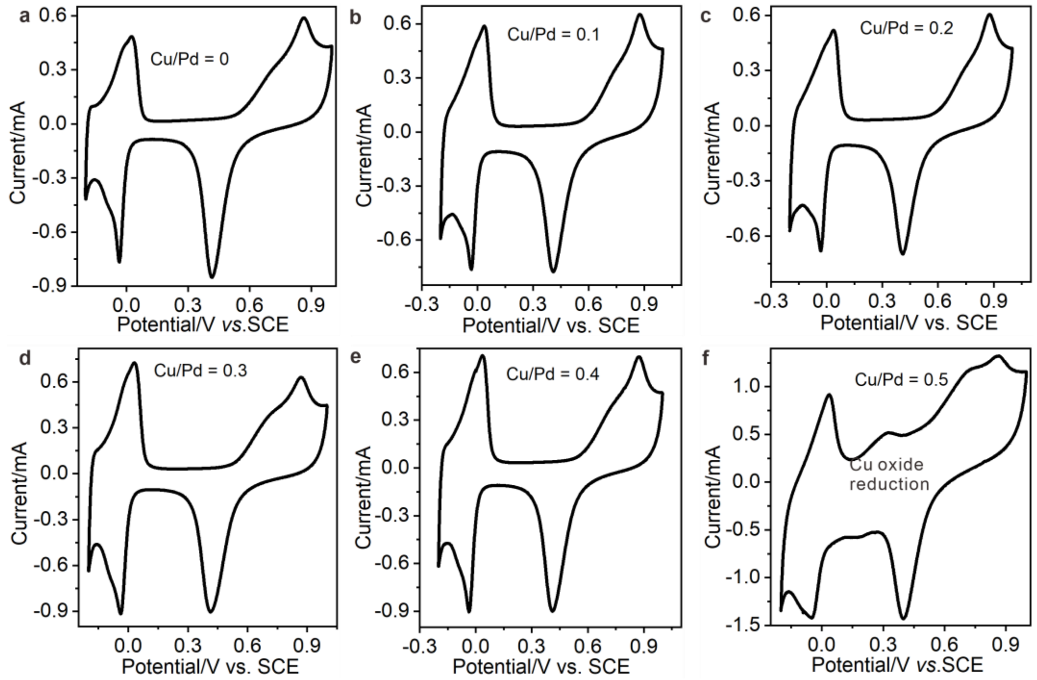

Supplementary Figure 14. CV curves of PdCu@Cu_2O with different Cu/Pd ratio. (a) Cu/Pd = 0. (b) Cu/Pd = 0.1. (c) Cu/Pd = 0.2. (d) Cu/Pd = 0.3. (e) Cu/Pd = 0.4. (f) Cu/Pd = 0.5.

Note: The Cu oxide reduction peak in CV was not observed under acidic conditions when Cu/Pd ratio ≤ 0.4, indicating there was no Cu^+ on the surface.

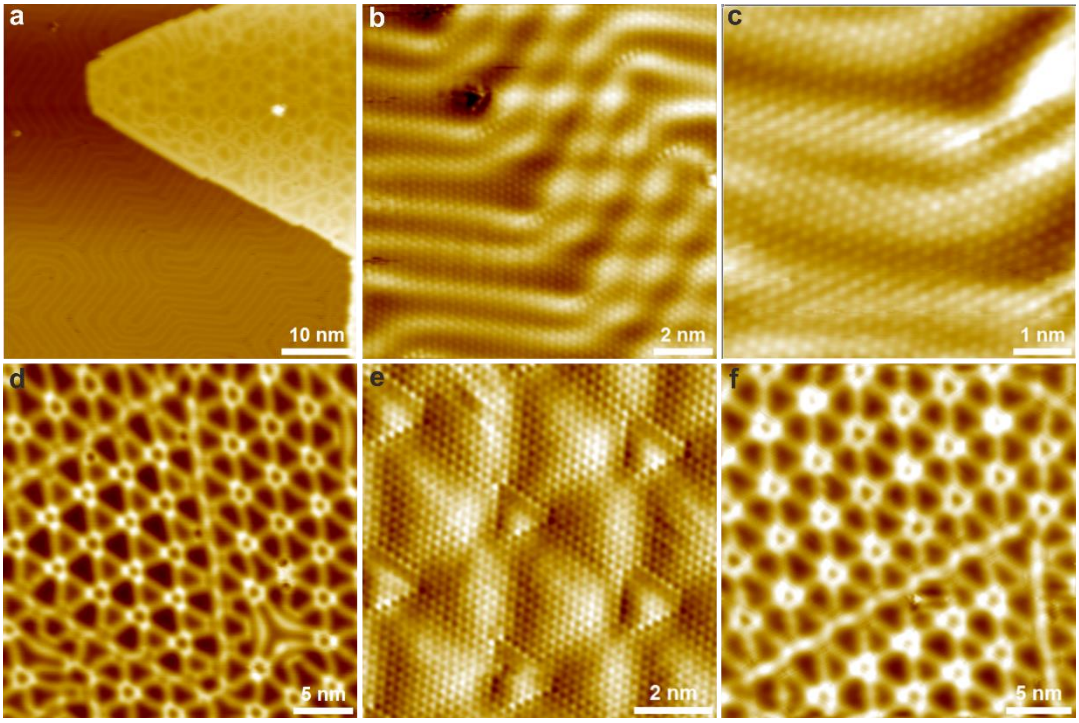

Supplementary Figure 15. STM images of Pd(111) with ~1.45 ML deposited Cu. (a) bilayer structure. (b, c) The resolution of first layer. (d-f) The resolution of second layer. Scanning parameters: (a) $V_s = 0.02$ V, $I = 3.0$ nA; (b) $V_s = 0.01$ V, $I = 5.1$ nA; (c) $V_s = 0.003$ V, $I = 5.1$ nA; (d) $V_s = 1.0$ V, $I = 0.5$ nA; (e) $V_s = 0.005$ V, $I = 3.9$ nA; (f) $V_s = 0.005$ V, $I = 3.9$ nA.

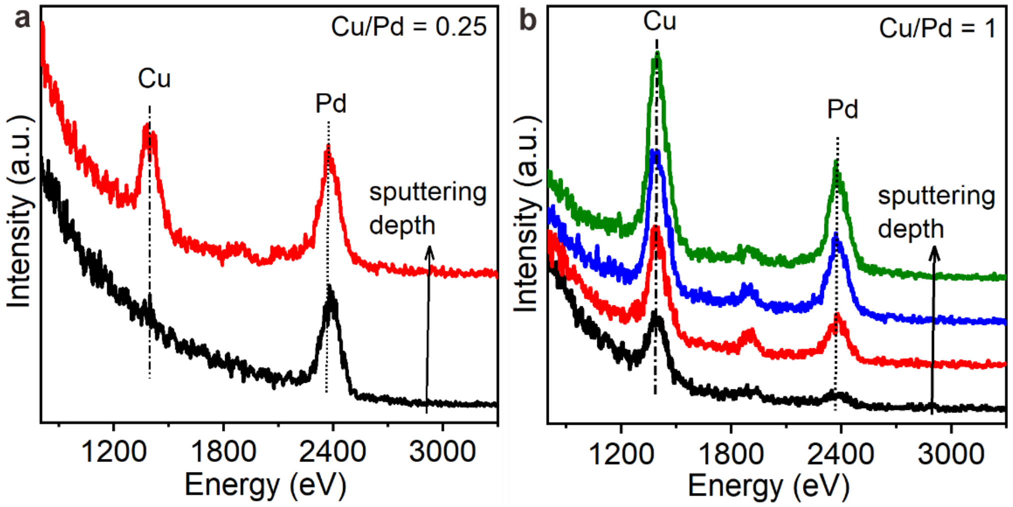

Supplementary Figure 16. High-sensitivity low energy ion scattering spectra (HS-LEISS) for the surface species of PdCu@Cu_2O with different Cu/Pd ratio. (a) Cu/Pd = 0.25, deposited Cu was less than 1 ML. (b) Cu/Pd = 1, deposited Cu was more than 1 ML.

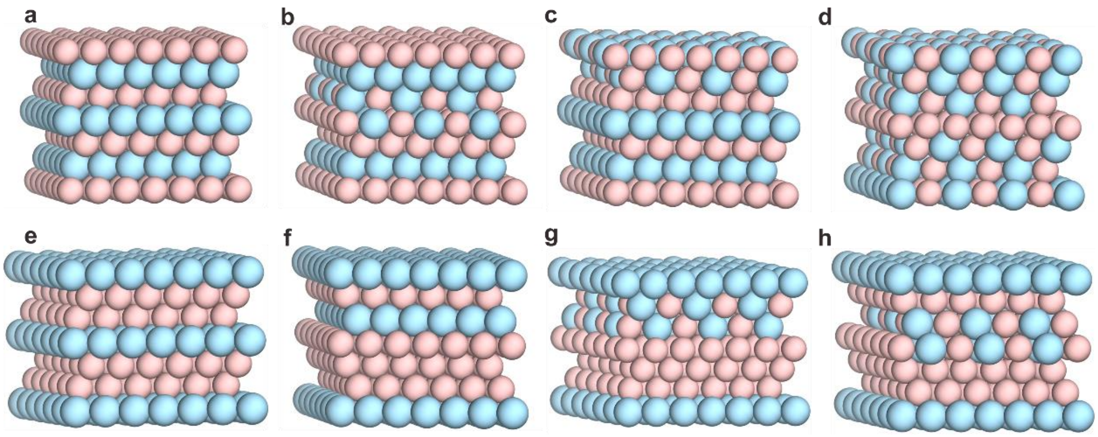

Supplementary Figure 17. Structures of different arrangement of Cu atoms on Pd(111) lattice with the ratio of Cu/Pd = 4/3 (a-h).

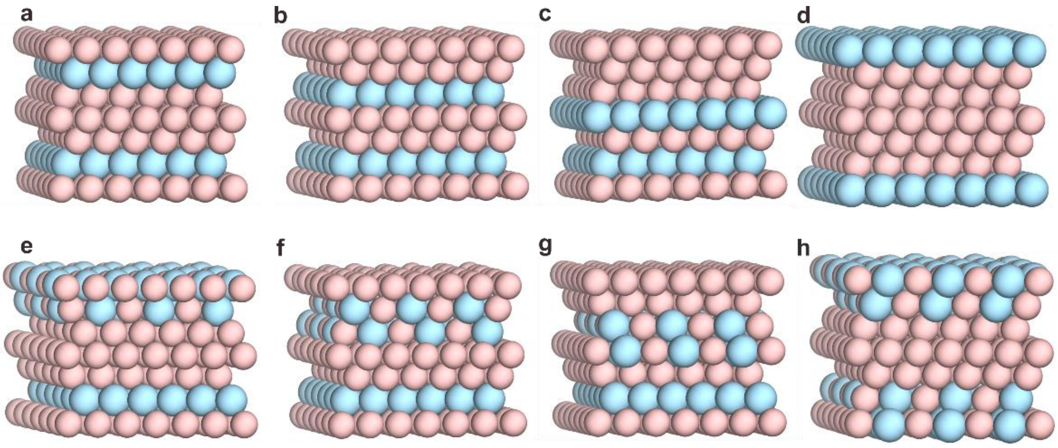

Supplementary Figure 18. Structures of different arrangement of Cu atoms on Pd(111) lattice with the ratio of Cu/Pd = 5/2 (a-h).

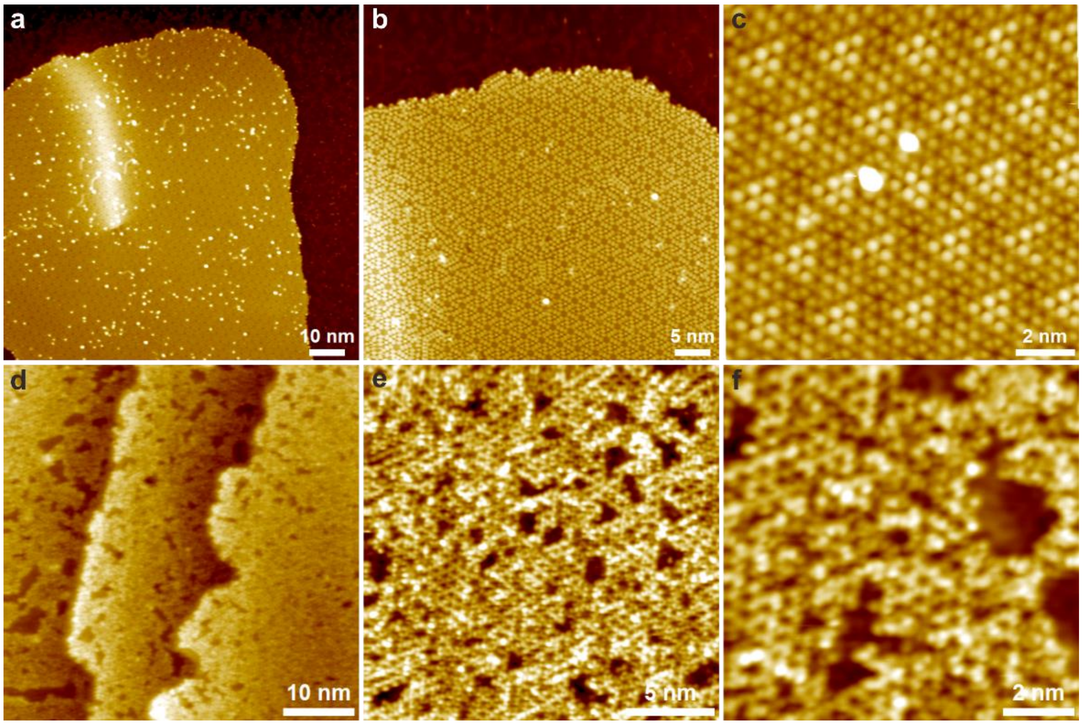

Supplementary Figure 19. STM images of the oxidized PdCu@Cu_2O. (a-c) 0.45 ML Cu_2O on Pd(111). (D-F) ~1.0 ML Cu_2O on PdCu. Scanning parameters: (a) V_s = 1.0 V, I = 0.1 nA; (b) V_s = -1.0 V, I = 0.1 nA; (c) V_s = -0.7 V, I = 0.5 nA; (d) V_s = -1.0 V, I = 0.1 nA; (e) V_s = -0.1 V, I = 0.5 nA; (f) V_s = -0.1 V, I = 3.0 nA.

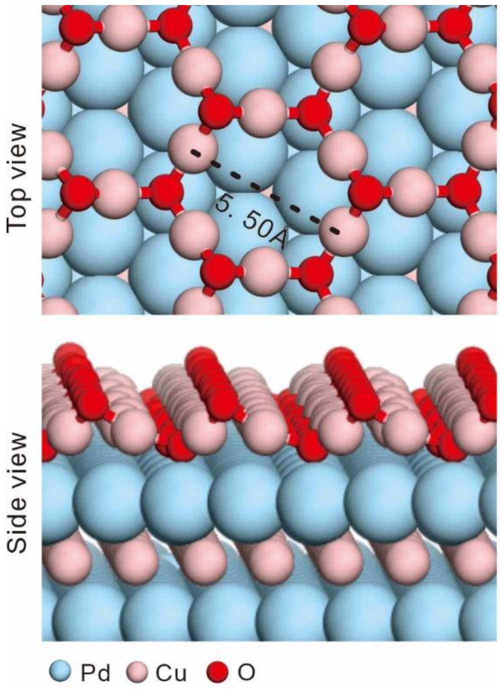

Supplementary Figure 20. Theoretical model for PdCu@Cu_2O surface.

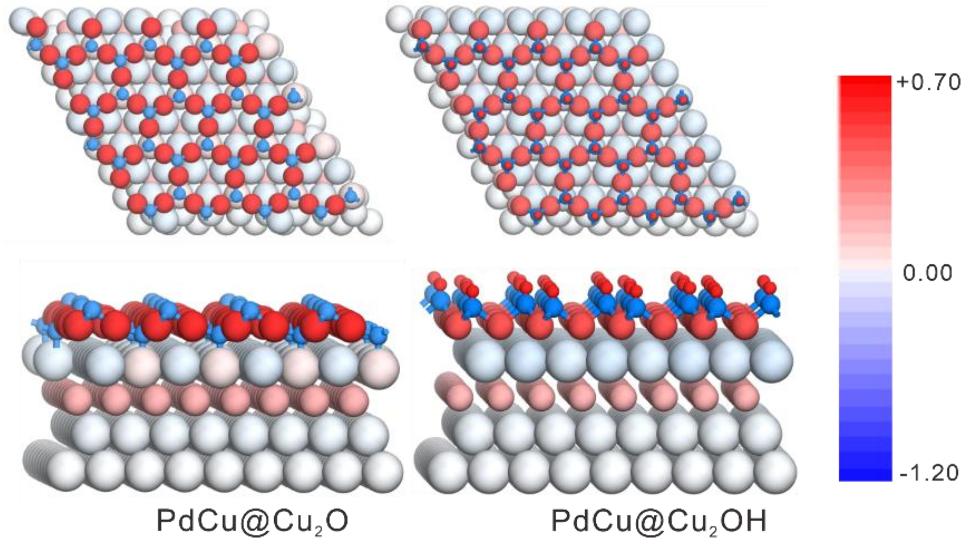

Supplementary Figure 21. Bader charge of PdCu@Cu_2O and PdCu@Cu_2OH models. Note: The Bader charge value of the Cu on the atomic layer Cu_2O structure of PdCu@Cu_2O was ~+0.58 a.u., which was similar to the value of Cu (~+0.50 a.u.) in the Cu_2O bulk. Compared the Bader charge of Cu atoms on PdCu@Cu_2O and PdCu@Cu_2OH surfaces, it was found that forming O-H bond would partially reduce the Cu of Cu_2O overlayer.

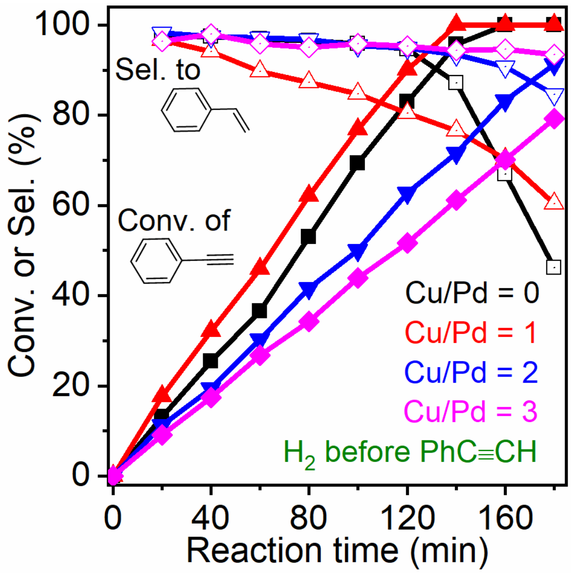

Supplementary Figure 22. Catalytic performance of semi-hydrogenation of PhC≡CH over PdCu@Cu_2O with different Cu/Pd ratio when H_2 was introduced before adding PhC≡CH. Reaction conditions: 10 mL ethanol; 2 μmol Pd; 4 mmol PhC≡CH (1:2,000); *T* = 303 K; pressure = 0.1 M Pa H_2 (H_2 treatment for 30 min before adding PhC≡CH).

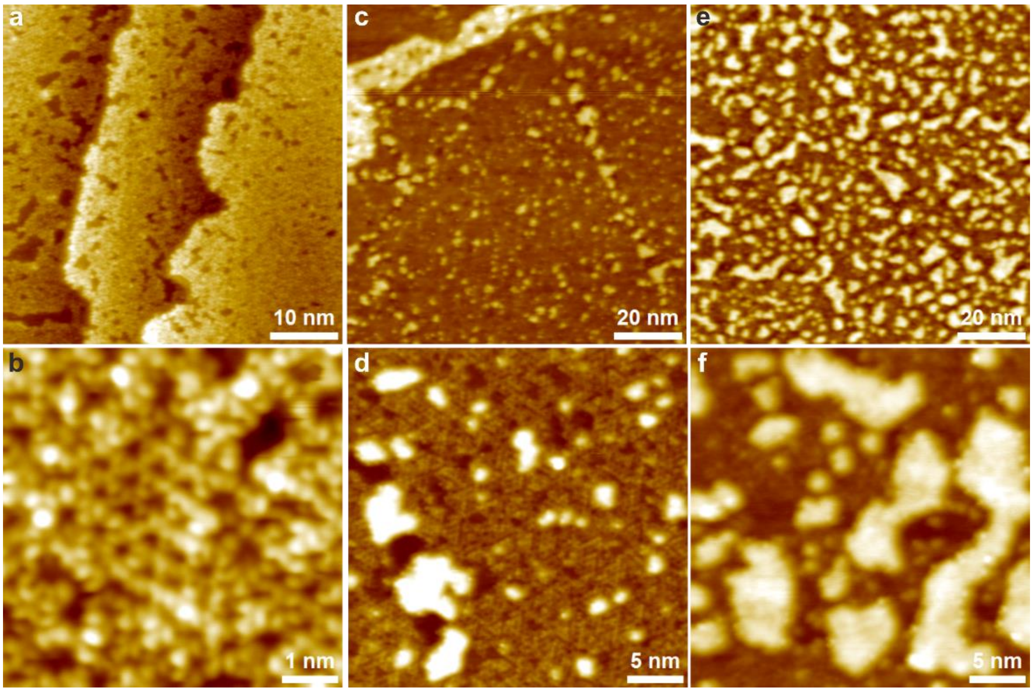

Supplementary Figure 23. Instability of PdCu@Cu_2O in H_2. (a, b) STM images of PdCu@Cu_2O with ~1.0 ML Cu_2O. (c, d) Exposure to 1.0×10^{-6} mbar H_2 at 300 K. (e, f) Exposure to 7.0×10^{-6} mbar H_2 at 300 K. Scanning parameters: (a) V_s = -1.0 V, I = 0.1 nA; (b) V_s = -0.2 V, I = 0.8 nA; (c) V_s = 1.0 V, I = 0.05 nA; (d) V_s = -0.2 V, I = 0.5 nA; (e) V_s = 1.0 V, I = 0.1 nA; (f) V_s = -0.2 V, I = 0.5 nA. Note: The surface Cu_2O with many defects was easily reduced to Cu by H_2.

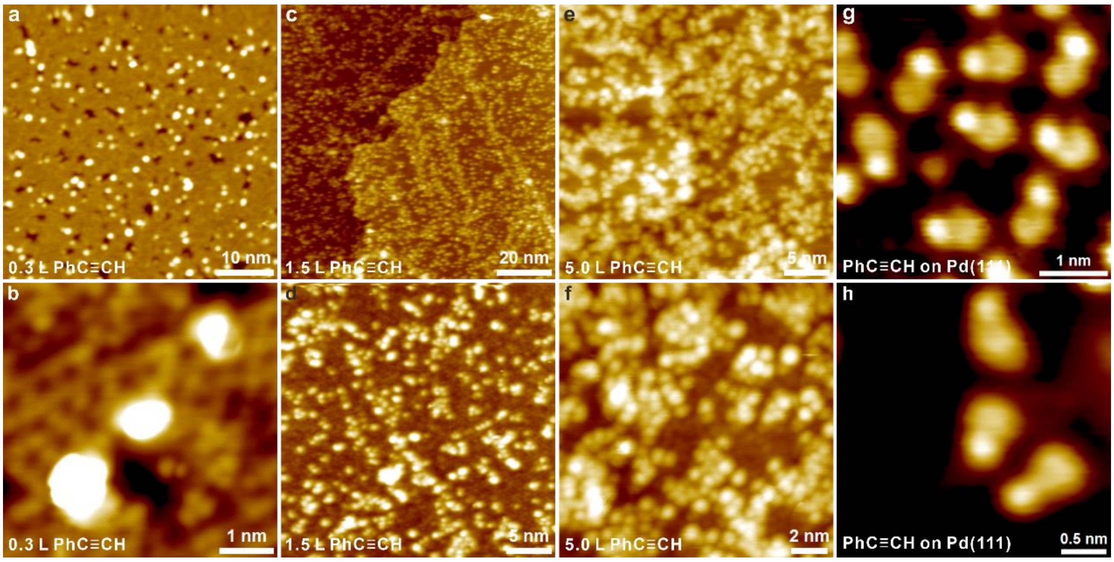

Supplementary Figure 24. STM characterizations of PhC≡CH adsorption on PdCu@Cu_2O and Pd(111). (a, b) 0.3 L PhC≡CH on PdCu@Cu_2O. (c, d) 1.5 L PhC≡CH on PdCu@Cu_2O. (e, f) 5.0 L PhC≡CH on PdCu@Cu_2O. (g, h) PhC≡CH on Pd(111). Scanning parameters: (a) V_s = -1.0 V, I = 0.2 nA; (b) V_s = -0.5 V, I = 0.3 nA; (c) V_s = 1.0 V, I = 0.05 nA; (d) V_s = -0.2 V, I = 0.5 nA; (e) V_s = 1.0 V, I = 0.1 nA; (f) V_s = -1.0 V, I = 0.1 nA.

Note: The defective Cu_2O of PdCu@Cu_2O could not be reduced in the presence of PhC≡CH. PhC≡CH on PdCu@Cu_2O appeared round-shape rather than pear-shape as those adsorbed on clean Pd(111), indicating the different structures adsorbed on two surfaces. At low coverage, PhC≡CH was discovered mainly adsorbed at the interface between Cu_2O and metal substrate.

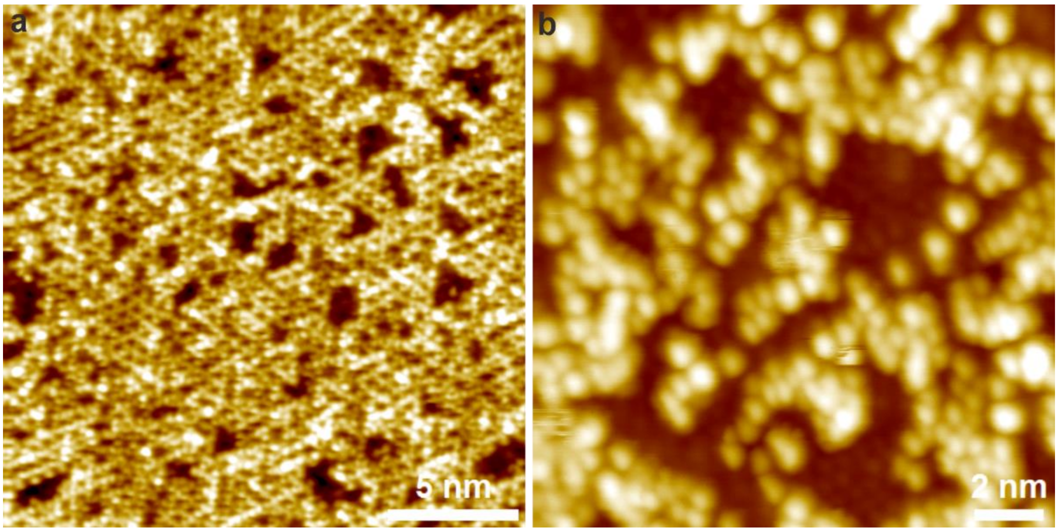

Supplementary Figure 25. STM characterizations of the stability of PdCu@Cu_2O-C≡CPh. (a) PdCu@Cu_2O. (b) PdCu@Cu_2O stabilized by adsorbed PhC≡CH in H_2. Scanning parameters: (a) V_s = -0.1 V, I = 0.5 nA; (b) V_s = 1.0 V, I = 0.1 nA.

Note: No reduction of Cu_2O with adsorbed PhC≡CH can be observed in $1E^{-6}$ mbar H_2. In comparison, PdCu@Cu_2O could be easily reduced by H_2 when there was no adsorption of PhC≡CH.

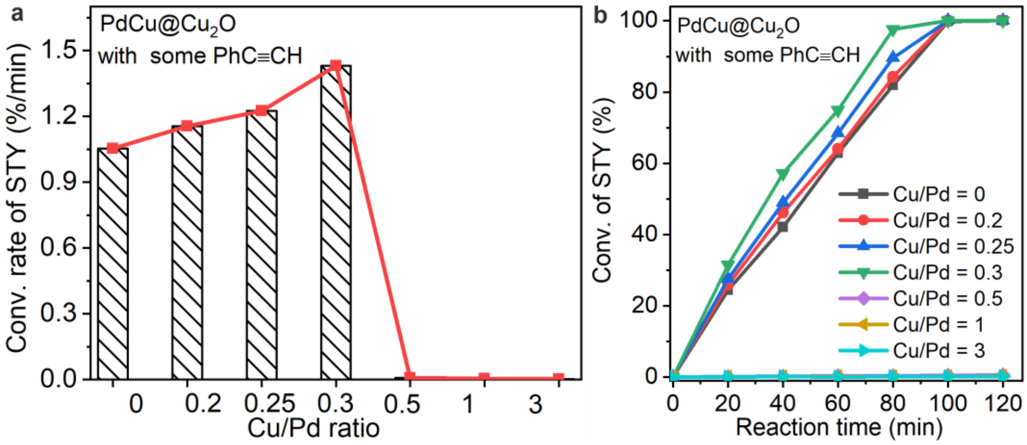

Supplementary Figure 26. Effect of PhC≡CH pre-treatment on the catalytic performance of styrene hydrogenation over PdCu@Cu_2O. (a) Conversion rate of styrene hydrogenation. (b) Time-dependent catalysis of styrene hydrogenation. Reaction conditions: 10 mL ethanol; 2 μmol Pd; 4 mmol Styrene (1:2,000); T = 303 K; pressure = 0.1 M Pa (Before styrene hydrogenation taken place, added 30 uL PhC≡CH and stirred with PdCu@Cu_2O for 30 minutes).

Note: For PdCu@Cu_2O with Cu/Pd ratio ≥ 0.5, once PhC≡CH was introduced, PdCu@Cu_2O would exhibit negligible activity for hydrogenation of styrene, consistent with the high selectivity of semi-hydrogenation of PhC≡CH.

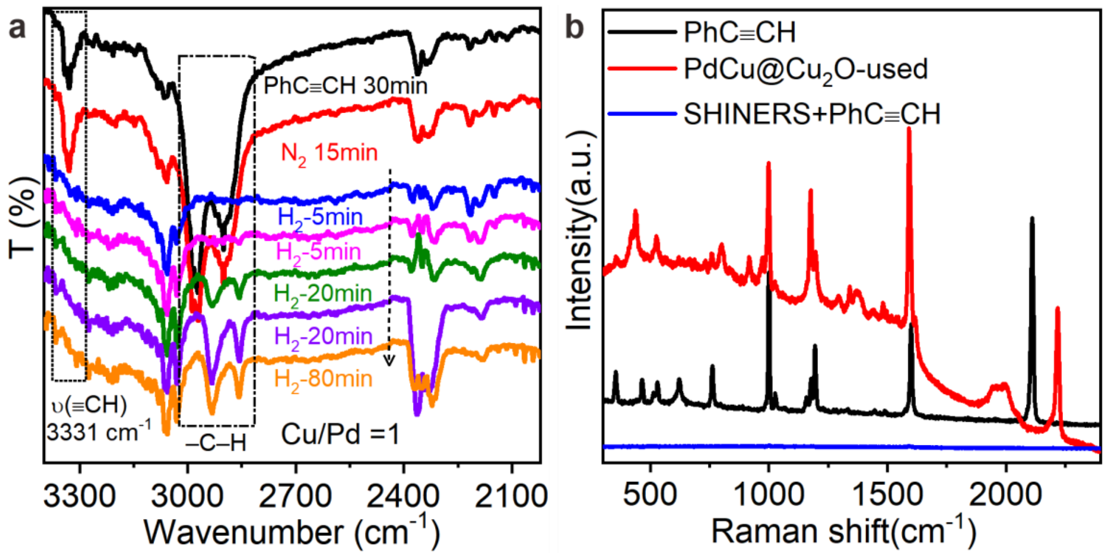

Supplementary Figure 27. (a) Monitoring the catalytic hydrogenation of PhC≡CH on PdCu@Cu_2O using *in situ* infrared spectroscopy. (b) Surface-enhanced Raman scattering (SERS) spectra of PdCu@Cu_2O after the semi-hydrogenation of PhC≡CH and references.

Note: There are two adsorption modes of PhC≡CH existed on the surface of PdCu@Cu_2O, namely molecular and dissociated modes. The signal of terminal C-H (υ(≡C-H)) disappeared when H_2 treatment was introduced, while the signal of alkynyl-copper (Cu(I)-C≡CPh) structure still exists even the H_2 treatment lasts for 80 min, indicating the PhC≡C group would be maintained upon hydrogenation.

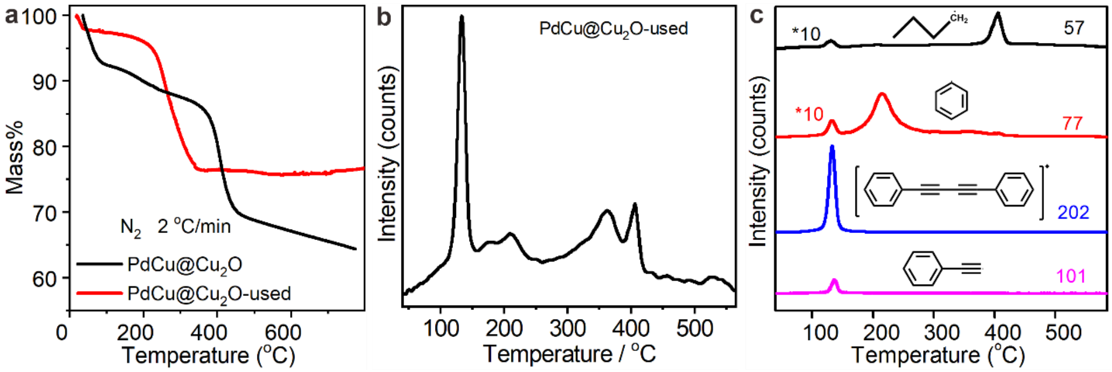

Supplementary Figure 28. (a) TGA of PdCu@Cu_2O (Cu/Pd = 1) before and after semi-hydrogenation of PhC≡CH. (b) The total ionization intensity *vs* temperature profile of PdCu@Cu_2O-used detected by TPD-MS. (c) Relative ionization intensities of the main decomposition products of PdCu@Cu_2O-used detected by TPD-MS.

Note: The mass loss of PdCu@Cu_2O-used (Cu/Pd = 1) was less than that of fresh PdCu@Cu_2O at a lower temperature, indicating the surface species of PdCu@Cu_2O had changed. In the results of TPD-MS, two major decomposition products [PhC≡C (m/z = 101) and $(PhC≡C)_2$ (m/z = 202)] were detected of PdCu@Cu_2O-used at ~145 ^{o}C.

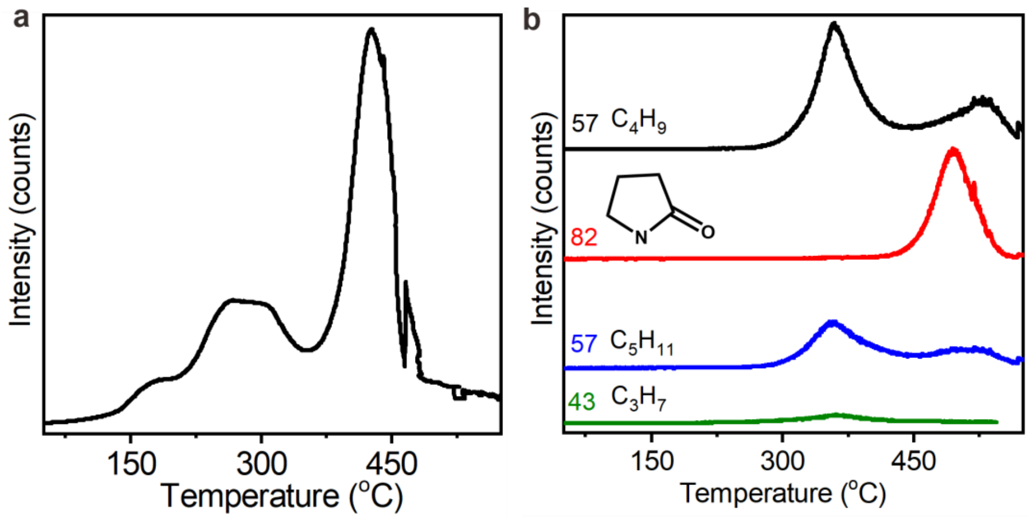

Supplementary Figure 29. *In situ* TPD-MS analysis of PdCu@Cu_2O. (a) The total ionization intensity *vs* temperature. (b) Relative ionization intensities of the main decomposition products *vs* temperature. Note: As demonstrated by TPD-MS, the decomposition of PdCu@Cu_2O was consistent with the standard spectrum of PVP, PhC≡C^- and $(PhC≡C)_2$ dimer were not observed.

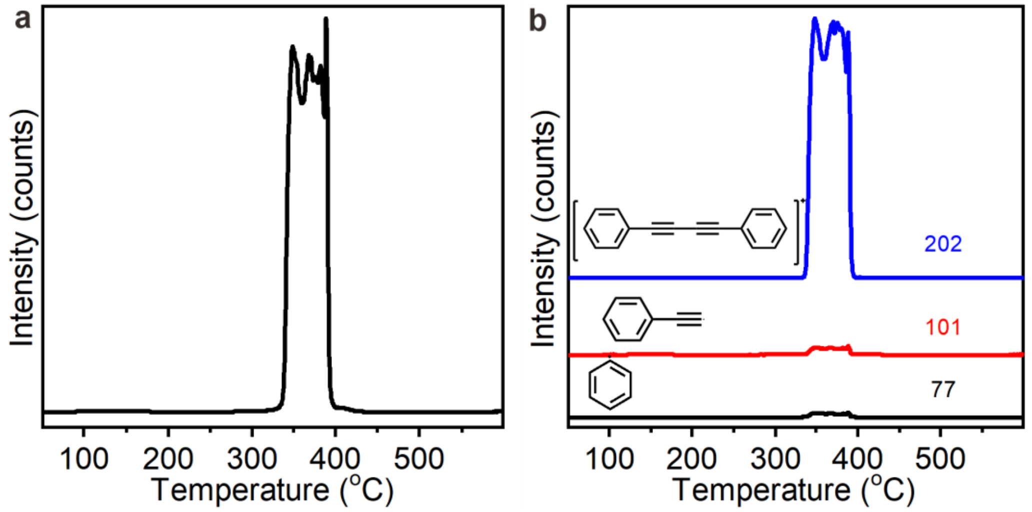

Supplementary Figure 30. *In situ* TPD-MS characterization of $(PhC≡C)_2$ dimer with electron ionization. (a) The total ionization intensity *vs* temperature. (b) Relative ionization intensities of the main decomposition products *vs* temperature. Note: The signal of $(PhC≡C)_2$ dimer was appeared from 350 °C, which was much higher than that of PdCu@Cu_2O-used. Thus, the new species on PdCu@Cu_2O-used was the dissociated PhC≡C^-, and the detected $(PhC≡C)_2$ dimer comes from the coupling of PhC≡C^- desorbed from PdCu@Cu_2O-used.

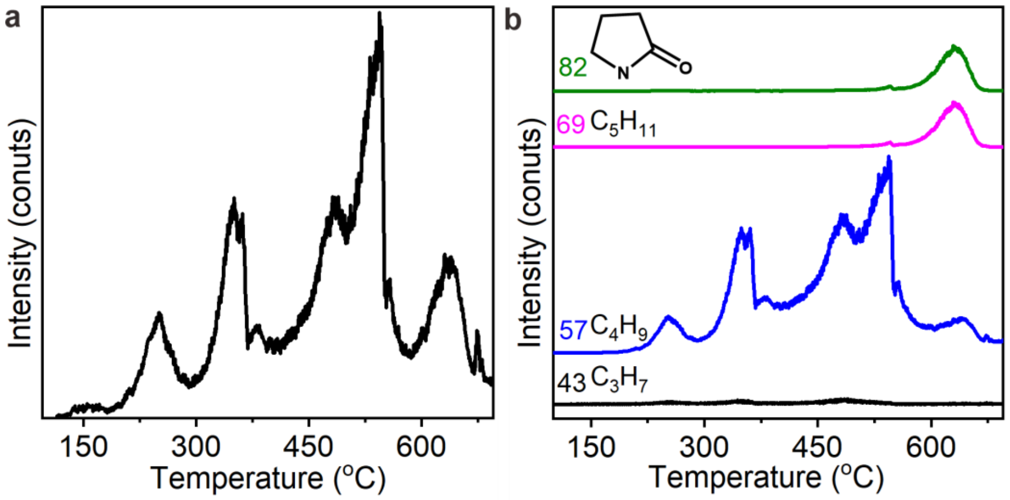

Supplementary Figure 31. *In situ* TPD-MS characterization of Pd NSs after reaction with electron ionization. (a) The total ionization intensity *vs* temperature. (b) Relative ionization intensities of the main decomposition products *vs* temperature. Note: The decomposition product of Pd NSs after reaction were PVP, no $(PhC{\equiv}C)_2$ dimer, indicating that the dissociated $PhC{\equiv}C^-$ on PdCu@Cu_2O-used was related to Cu.

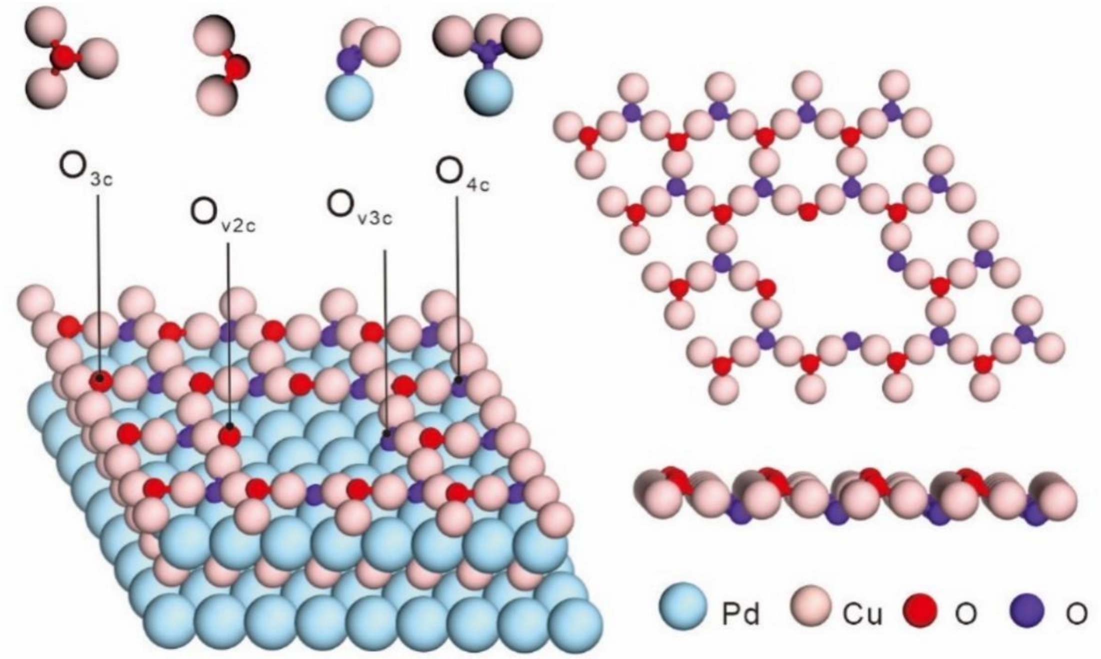

Supplementary Figure 32. The structures of different coordinated O sites on the defect-rich PdCu@Cu_2O surface. Note: There are four kinds of O sites on Cu_2O overlayer, including the four-coordinate O (O_{4c}) and three-coordinate O (O_{3c}) on the Cu_2O overlayer; and three-coordinate O and two-coordinate O adjacent to the defected sites, which are labelled as O_{v3c} and O_{v2c}.

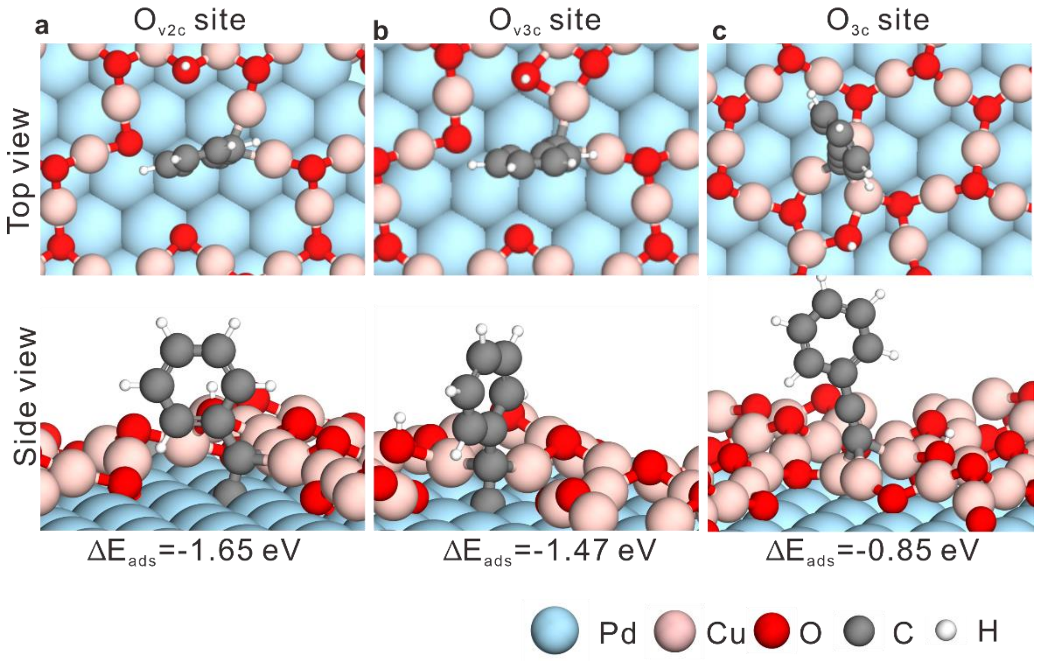

Supplementary Figure 33. Dissociated adsorption of PhC≡CH on different O sites (a-c). Note: The dissociated adsorption of PhC≡CH on the O_{v2c} and O_{v3c} sites were predicted to be exothermic by 1.65 eV and 1.47 eV, respectively, while on O_{3c} site, a relative weak adsorption energy of -0.85eV was predicted.

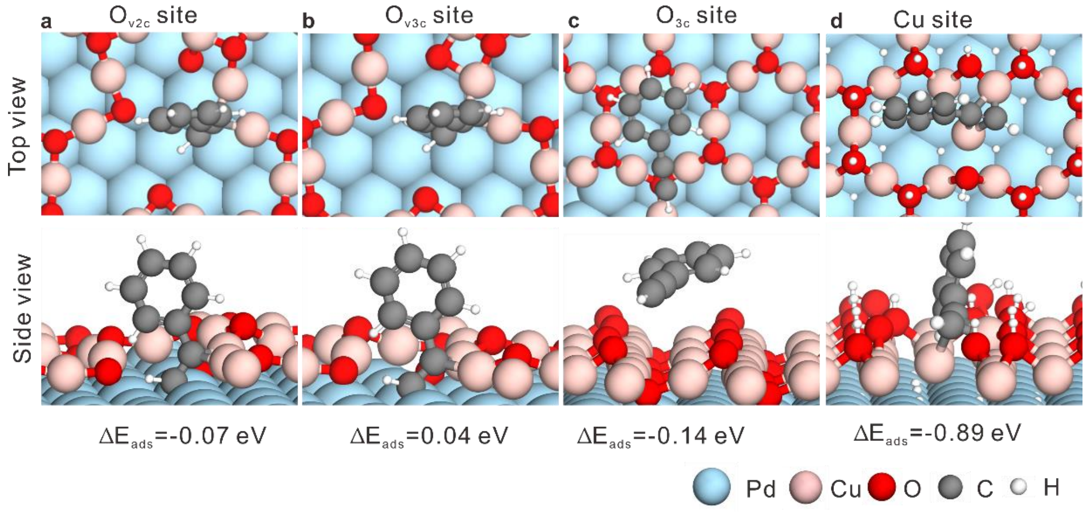

Supplementary Figure 34. Molecular adsorption of PhC≡CH on different O sites (a-c), and on the OH covered surface (d). Note: Molecular adsorptions were energetically less favorable than their dissociated counterparts.

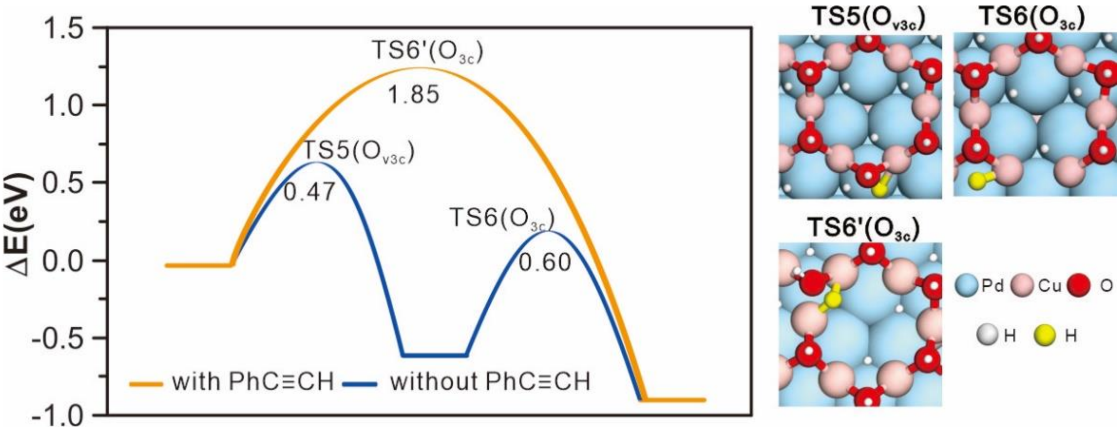

Supplementary Figure 35. Reaction barriers for OH removal at different sites. Note: It was found that the OH groups originated from O_{v3c} and O_{v2c} could be readily reduced to H_2O with low energy barriers (~ 0.47 eV and ~0.60 eV). When the PhC≡CH was introduced, the O_{v3c} and O_{v2c} sites could be passivated by PhC≡C group, which protected the OH group of Cu_2O overlayer from reductive removal.

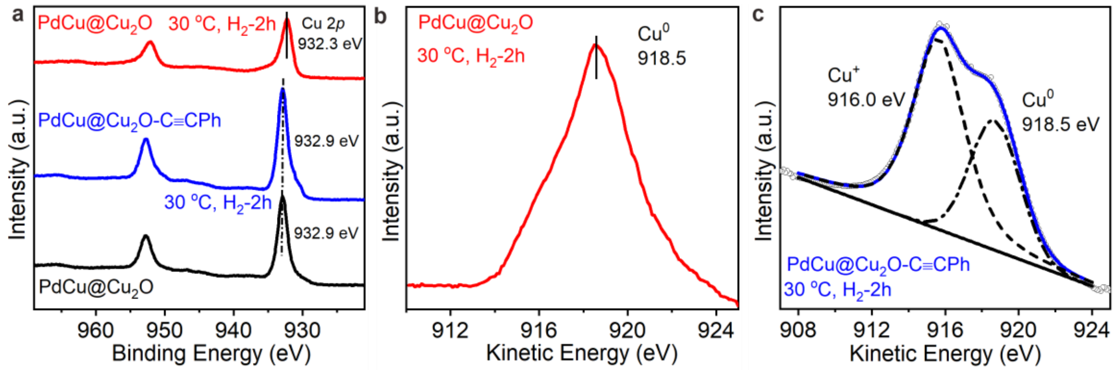

Supplementary Figure 36. The XPS spectra of Cu in PdCu@Cu_2O and PdCu@Cu_2O-C≡CPh (Cu/Pd = 1) which was subjected to H_2 treatment at 30 °C for 2h. (a) Cu 2p XPS. (b) Cu LMM XAES spectra in PdCu@Cu_2O after H_2 treatment at 30 °C for 2h. (c) Cu LMM XAES spectra in PdCu@Cu_2O-C≡CPh after H_2 treatment at 30 °C for 2h.

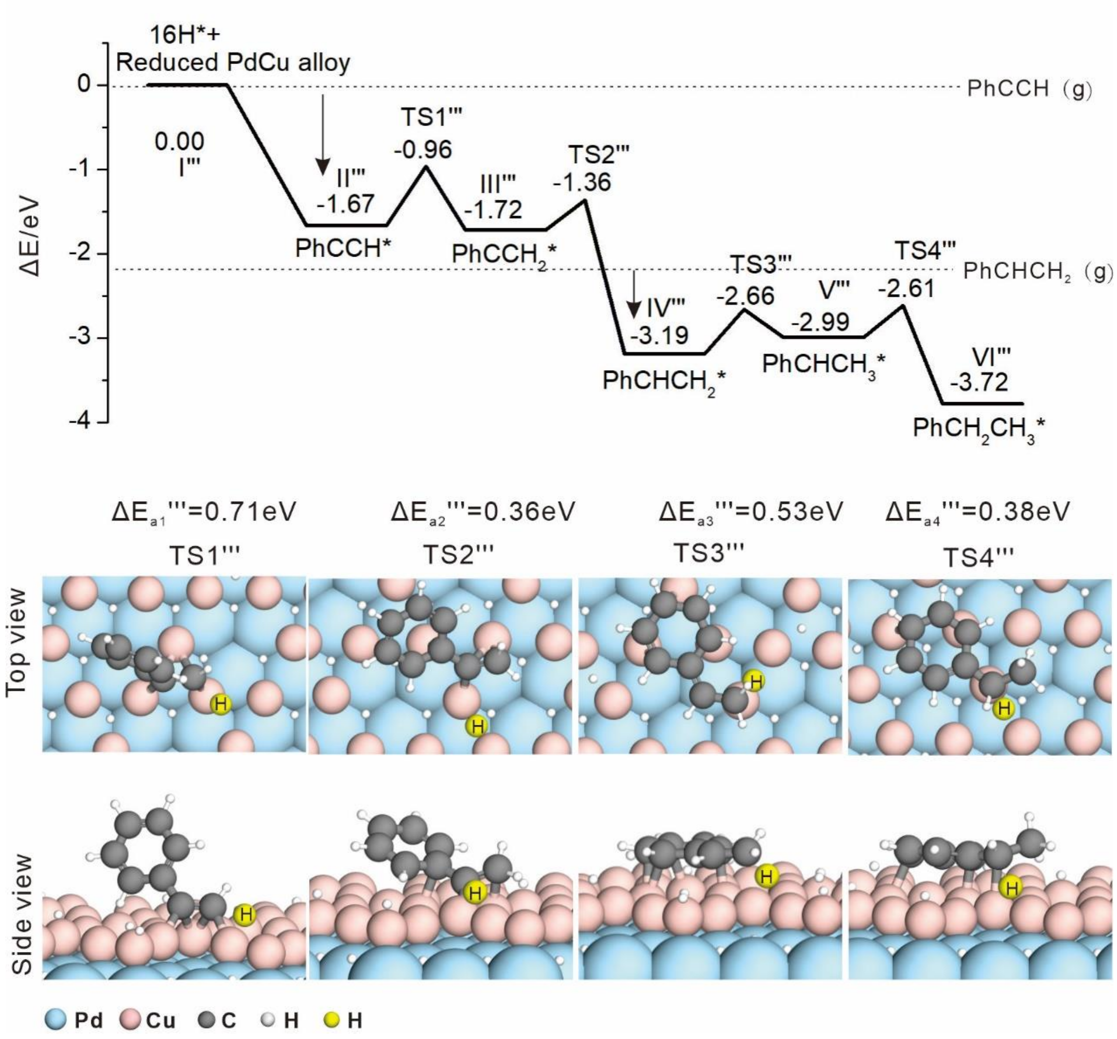

Supplementary Figure 37. Energy profile and optimized structures of the transition states of stepwise hydrogenation of PhC≡CH on the reduced PdCu alloy. Note: The Pd-Cu alloy model was built by simply removing all O atoms from PdCu@Cu_2O surface.

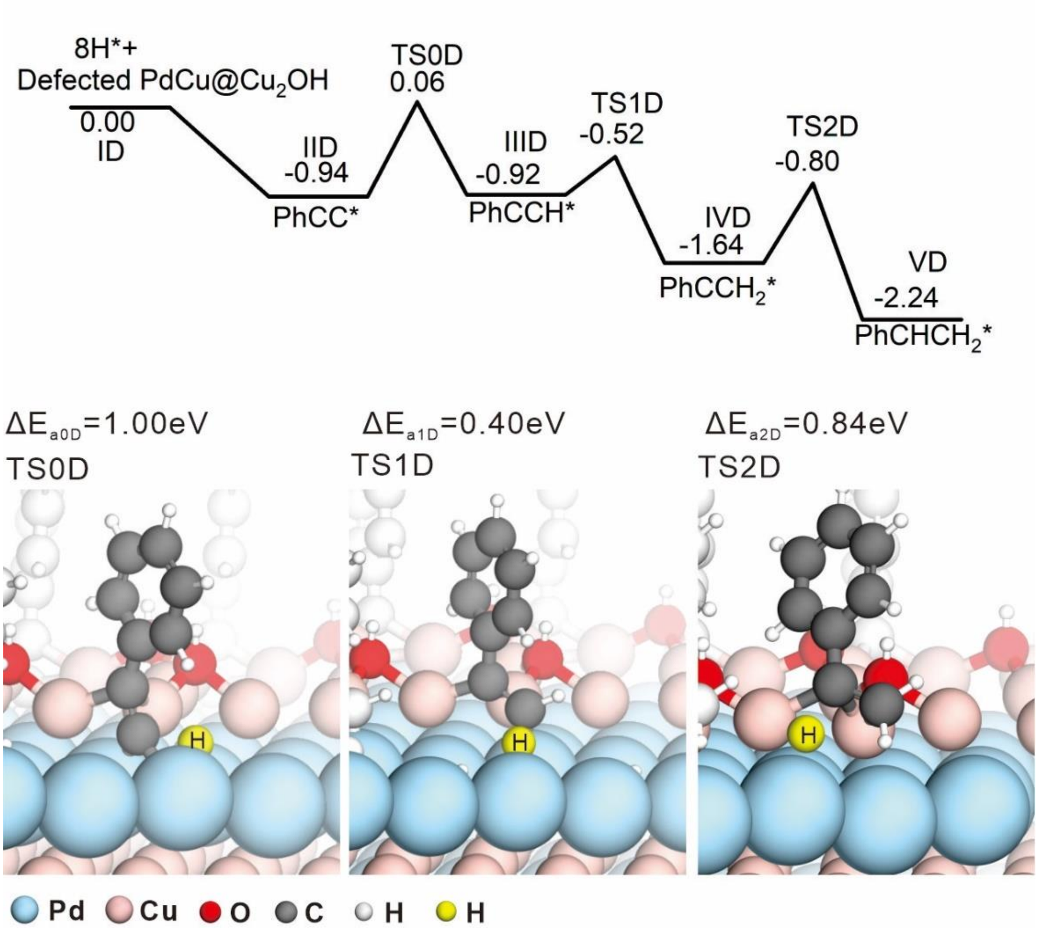

Supplementary Figure 38. Energy profile and optimized structures of the transition states of stepwise hydrogenation of the dissociated PhC≡CH adsorbed on the defected sites of PdCu@Cu_2O surface. Note: The hydrogenation barrier for Cu(I)-C≡CPh was predicted to be 1.0 eV, indicating that $PhC≡C^-$ at the boundary or defected site would only serve as spectator upon hydrogenation.

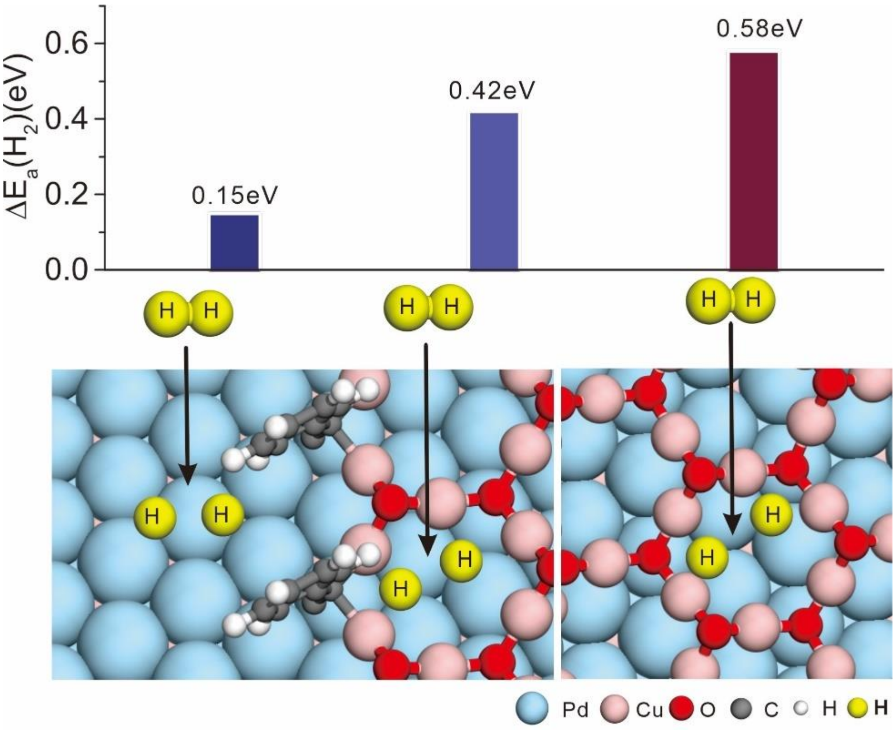

Supplementary Figure 39. The dissociation of H_2 on different Pd sites of PdCu@Cu_2O.

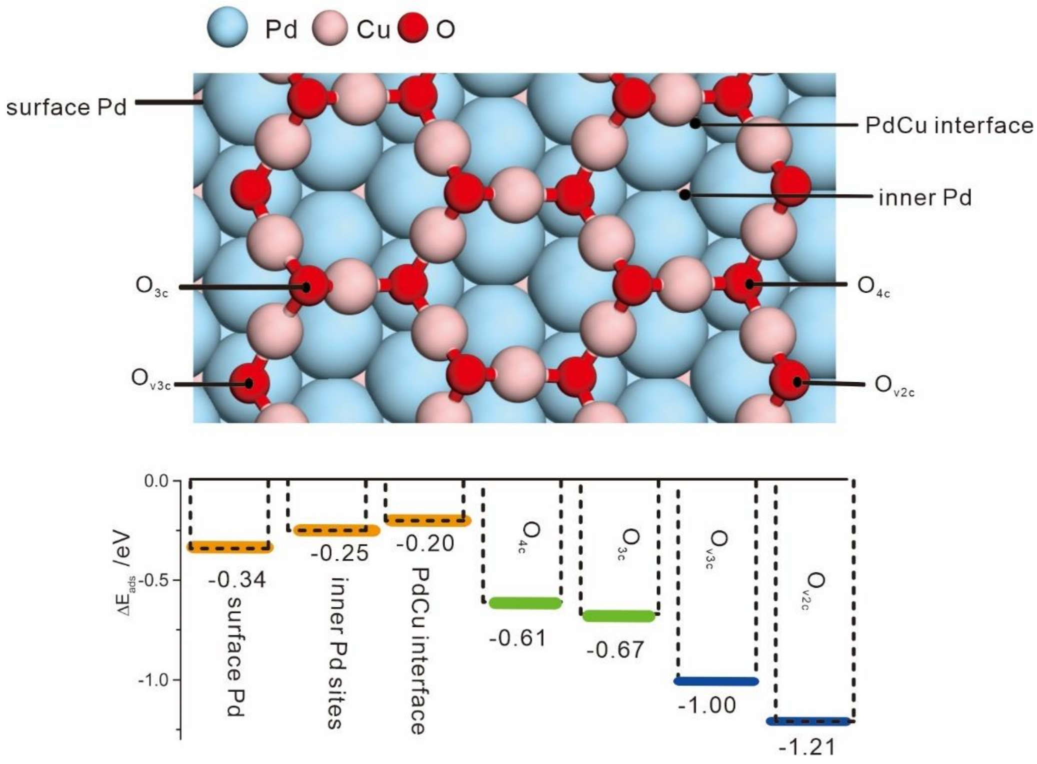

Supplementary Figure 40. The adsorption energies of H atoms on different sites of PdCu@Cu_2O surface.

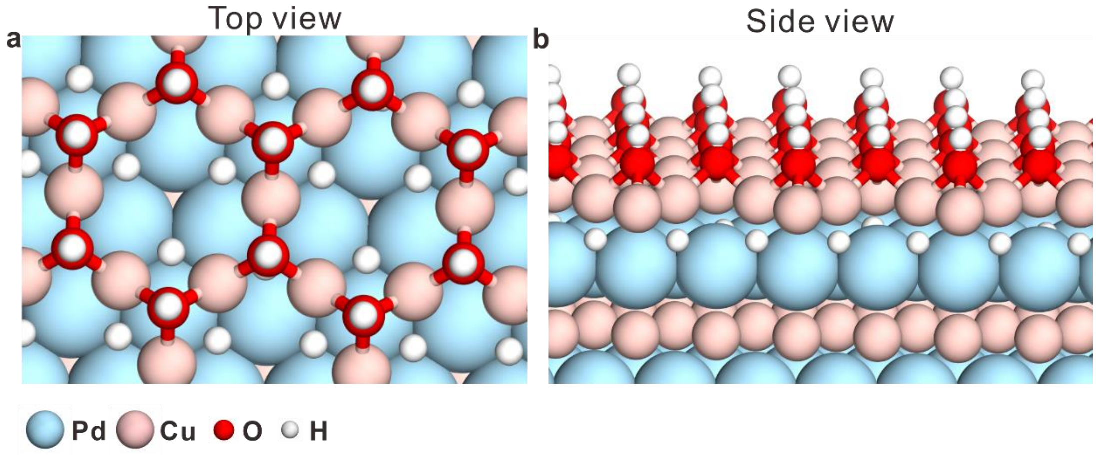

Supplementary Figure 41. The PdCu@Cu_2OH surface with H atoms attached to the surface O of PdCu@Cu_2O. (a) Top view. (b) Side view.

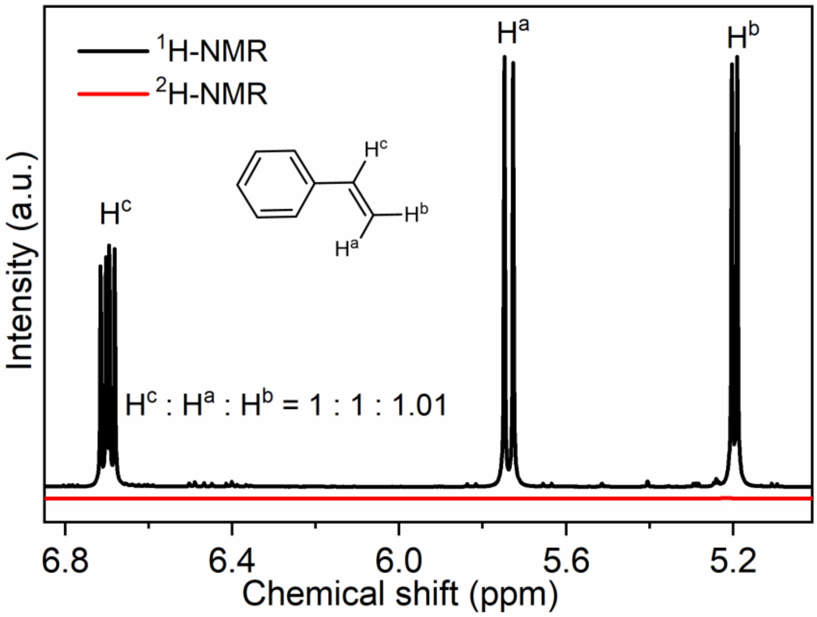

Supplementary Figure 42. H-NMR data for the products of PhC≡CH hydrogenation carried out on PdCu@Cu_2O-D_2 in CH_3OH as the solvent and under H_2 atmosphere (PdCu@Cu_2O was treated by D_2 for 30 min before hydrogenation). Note: No signal of β-deuterated product was observed, which indicating the $D^{\delta+}$ in O-$D^{\delta+}$ was not involvement in the hydrogenation.

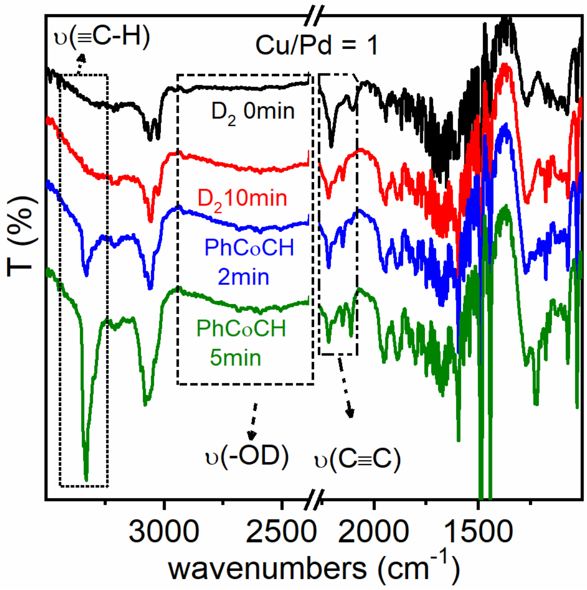

Supplementary Figure 43. *In situ* infrared spectroscopy of monitoring the catalytic hydrogenation process on PdCu@Cu_2O-C≡CPh with D_2 and PhC≡CH introduced successively. After the introduction of PhC≡CH, the signal of -OD remains unchanged, which also indicating the $D^{\delta+}$ in O-$D^{\delta+}$ was not involvement in the hydrogenation.

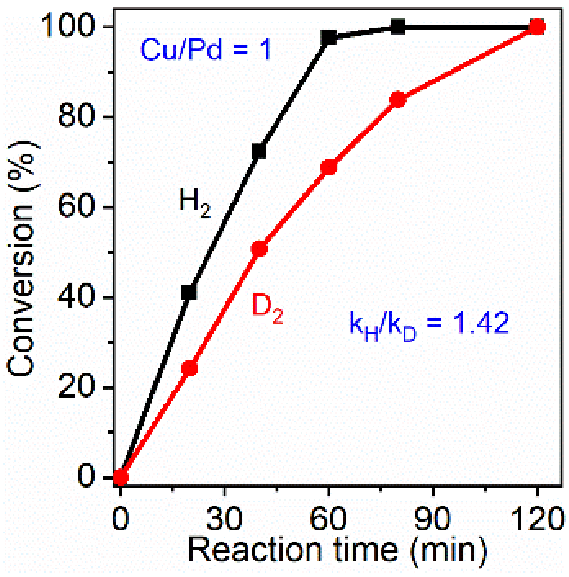

Supplementary Figure 44. Primary isotope effect observed on PdCu@Cu_2O (Cu/Pd = 1) in the semi-hydrogenation of PhC≡CH. Reaction conditions: 10 mL ethanol; 2 μmol Pd; 4 mmol PhC≡CH (1:2,000); T = 303 K; pressure = 0.1 MPa H_2. Note: A small kinetic isotope effect of 1.42 was observed, which confirmed the proton transfer not involved in the semi-hydrogenation of PhC≡CH.

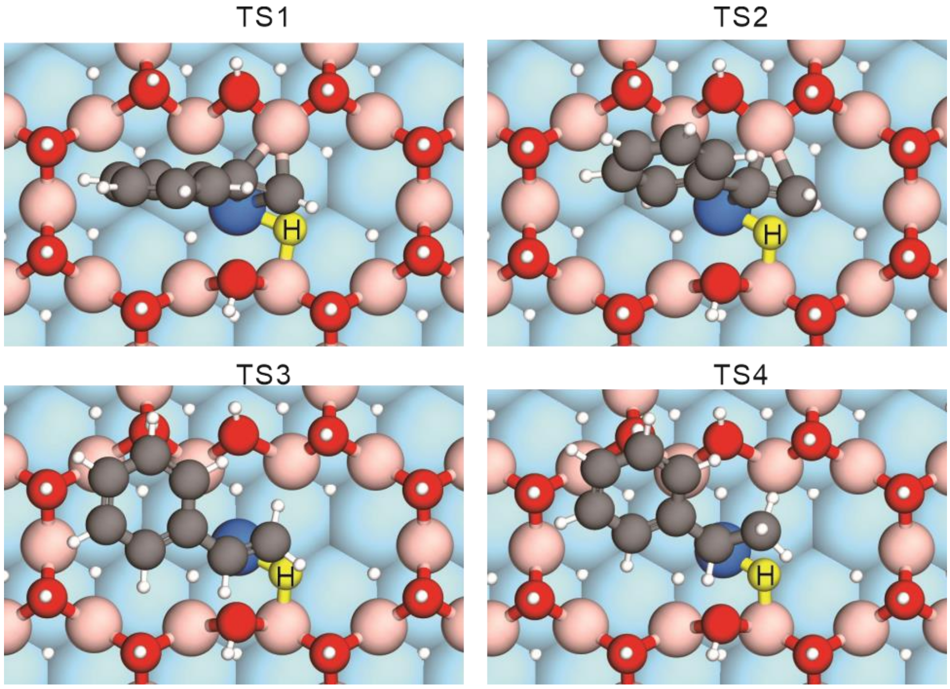

Supplementary Figure 45. Top view of the optimized structures of stepwise hydrogenation transition states of PhC≡CH on the PdCu@Cu_2O surface.

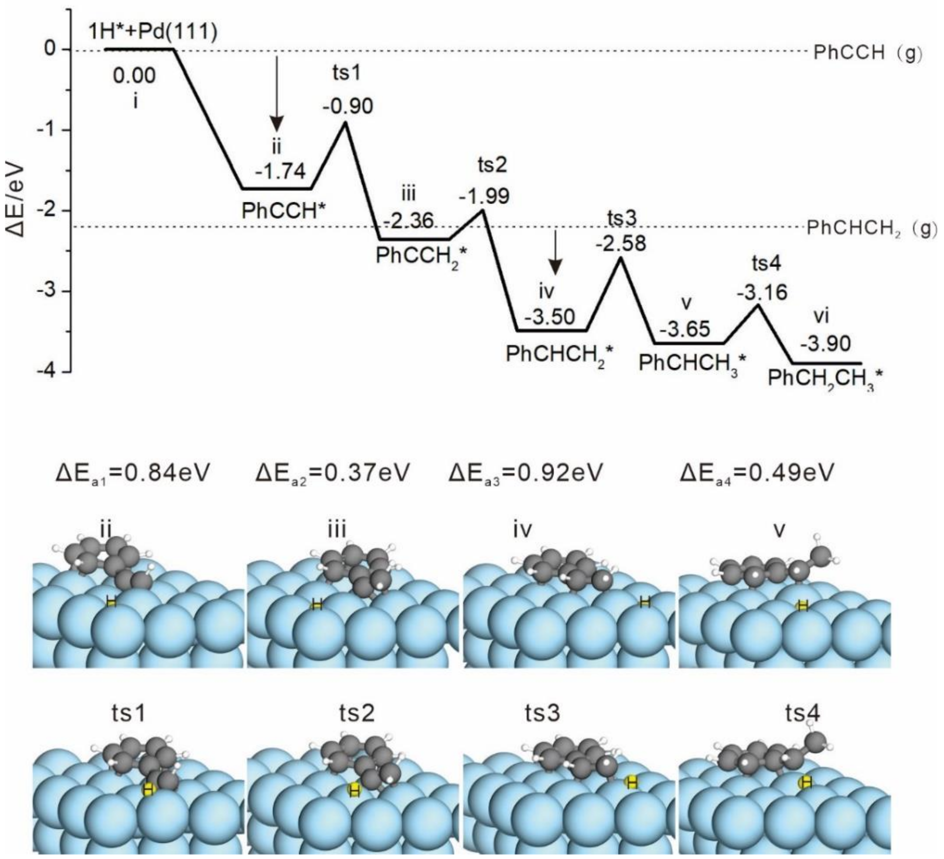

Supplementary Figure 46. Energy profile and optimized structures of the transition states of stepwise hydrogenation of PhC≡CH on the Pd surface.

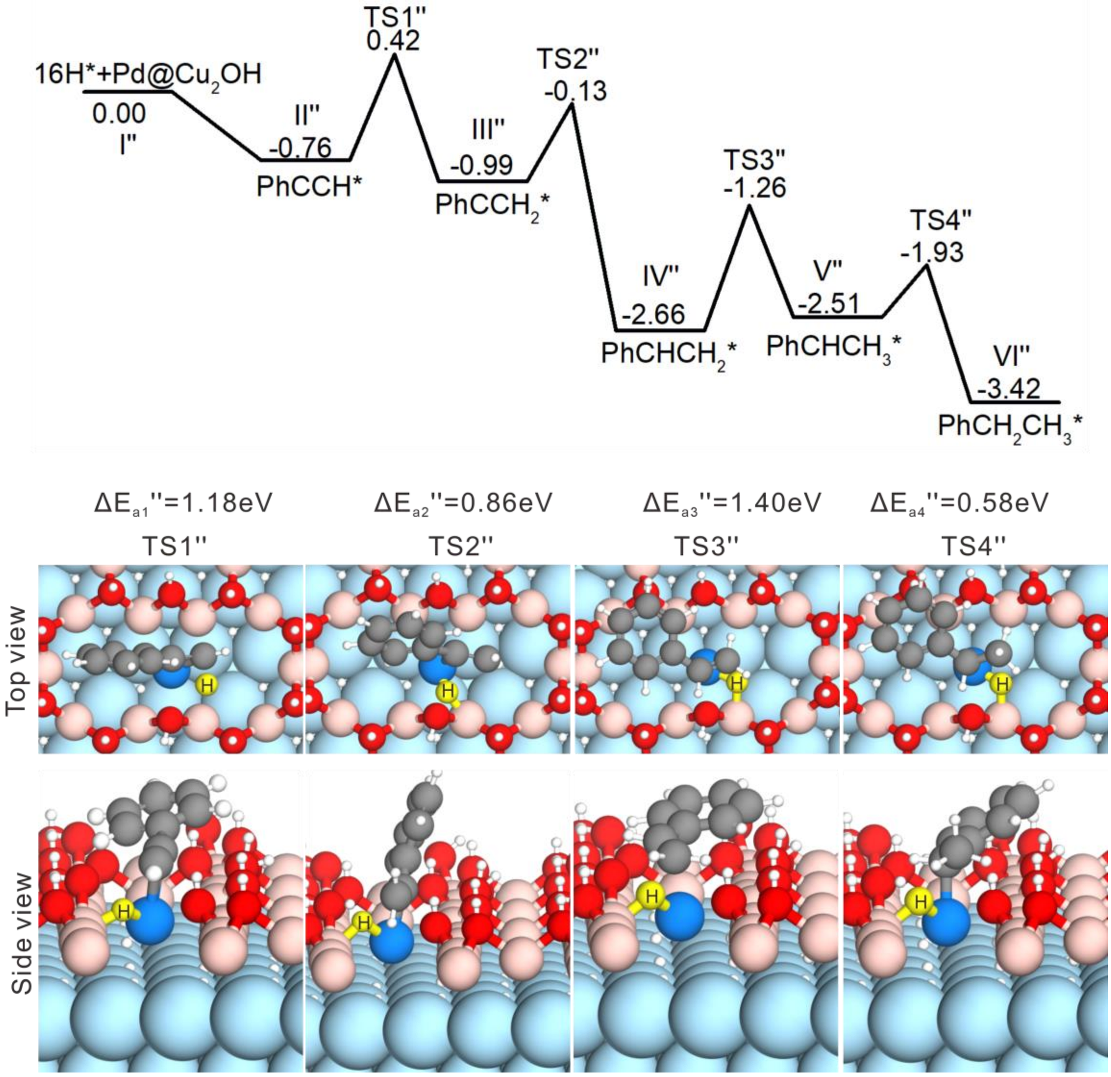

Supplementary Figure 47. Energy profile and optimized structures of the transition states of stepwise hydrogenation of PhC≡CH on the Pd@Cu_2O surface.

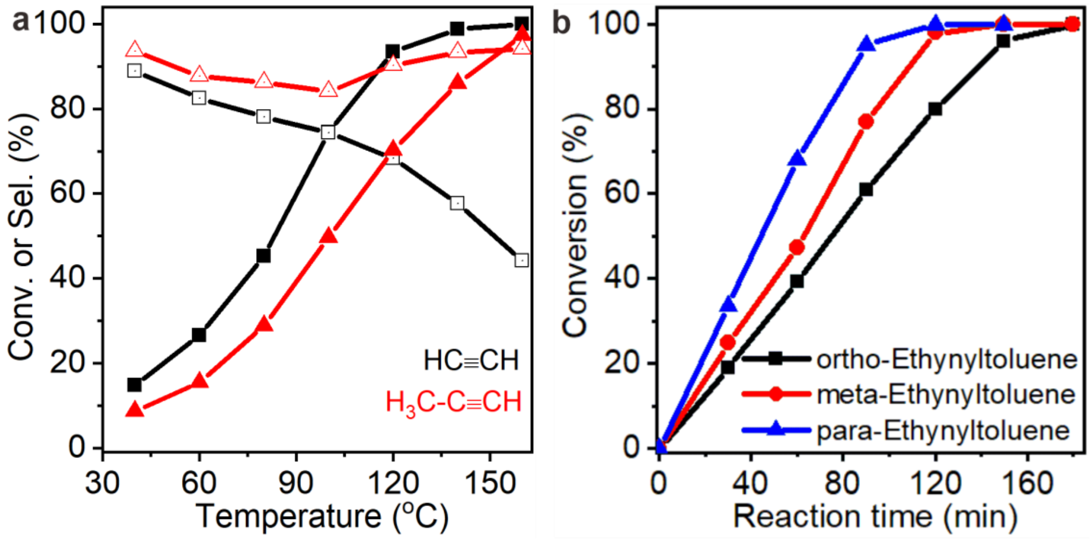

Supplementary Figure 48. The catalytic performance of $PdCu@Cu_2O$ depends on the alkyne size and triple bond position. (a) Gas phase hydrogenation of alkyne in a large excess of alkene over $PdCu@Cu_2O$ (Cu/Pd=1). Reaction condition: a gas mixture with a space velocity of 60,000 ml $h^{-1}g^{-1}$ was introduced into the reactor, simulating the front-end hydrogenation conditions with 1.0 vol.% C_2H_2 (or C_3H_4), 20.0 vol.% H_2 and 20.0 vol.% C_2H_4 (or C_3H_6), balanced with N_2. (b) The difference in catalytic performance of $PdCu@Cu_2O$ (Cu/Pd =1) caused by the triple bond position. Reaction conditions: 10 mL ethanol; 2 μmol Pd; 4 mmol alkyne; 303 K; 0.1 MPa H_2.

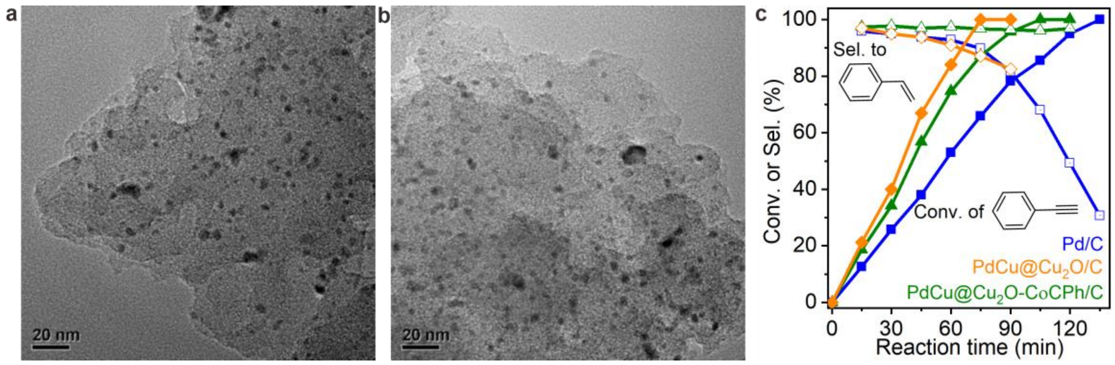

Supplementary Figure 49. Optimization of Commercial Pd Catalysts via Cu(I)-C≡CPh structure. Representative TEM images of (a) Pd/C and (b) $PdCu@Cu_2O$-C≡CPh/C. Catalytic performance of semi-hydrogenation of PhC≡CH catalyzed by (c) $PdCu@Cu_2O$-C≡CPh/C and references. Reaction conditions: 10 mL ethanol; 2 μmol Pd; 4 mmol PhC≡CH (1:2,000); T = 303 K; pressure = 0.1 MPa H_2 (For $PdCu@Cu_2O$/C, H_2 was introduced before PhC≡CH; for $PdCu@Cu_2O$-C≡CPh/C, PhC≡CH was introduced before H_2).

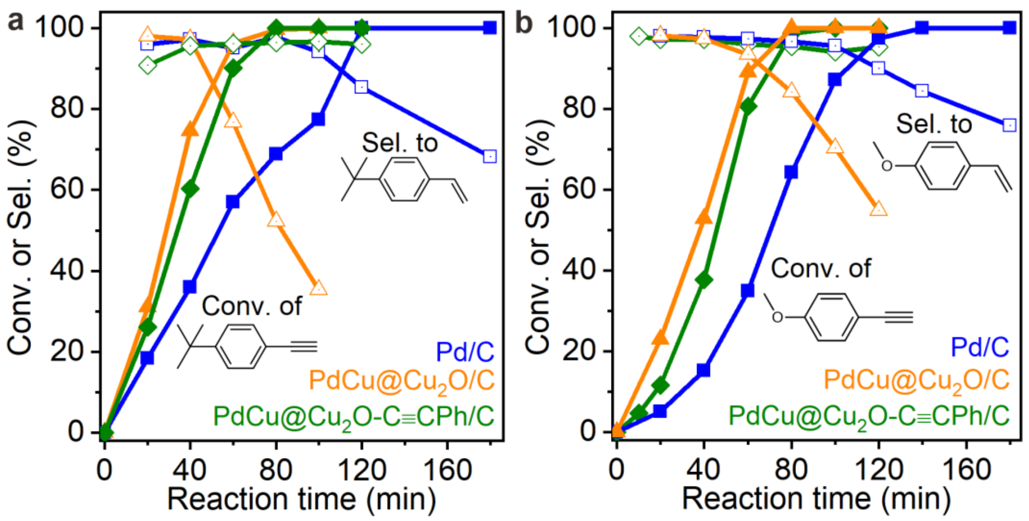

Supplementary Figure 50. Catalytic performance of semi-hydrogenation with an electron-donating group or an electron-withdrawing group of PhC≡CH with PdCu@Cu_2O-C≡CPh/C and references. (a) 4-tert-Butylphenylacetylene. (b) 4-Ethynylanisole. Reaction conditions: 10 mL ethanol; 2 μmol Pd; 4 mmol alkynes (1:2,000); T = 303 K; pressure = 0.1 MPa H_2 (For PdCu@Cu_2O/C, H_2 was introduced before substrate; for PdCu@Cu_2O-C≡CPh/C, PhC≡CH was introduced before the hydrogenation reaction takes place).

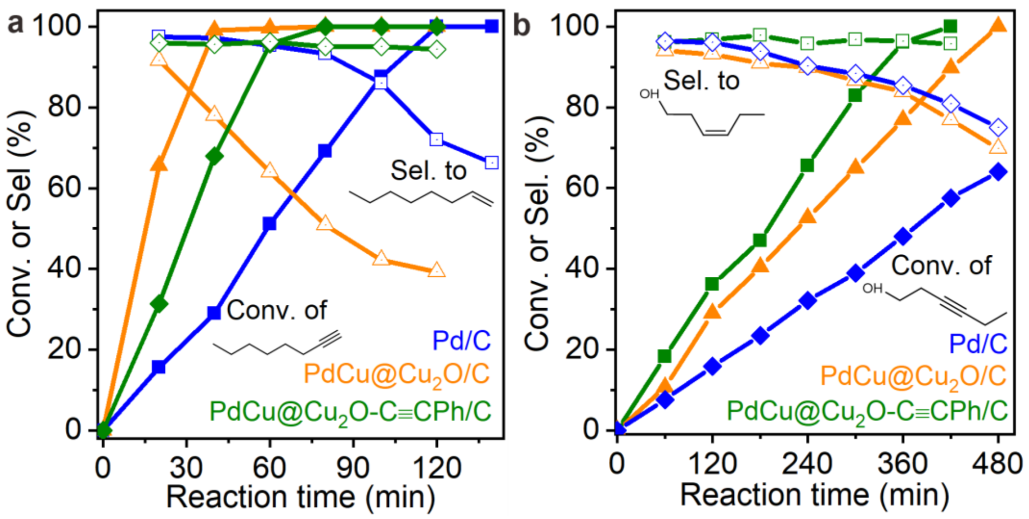

Supplementary Figure 51. Catalytic performance of hydrogenation of long carbon chain alkynes with PdCu@Cu_2O-C≡CPh/C and references. (a) 1-octyne. (b) 3-hexyn-1-olcas. Reaction conditions: 10 mL ethanol; 2 μmol Pd; 4 mmol alkynes (1:2,000, but for 3-hexyn-1-olcas was 1:500); T = 303 K; pressure = 0.1 MPa H_2 (For PdCu@Cu_2O/C, H_2 was introduced before substrate; for PdCu@Cu_2O-C≡CPh/C, PhC≡CH was introduced before the hydrogenation reaction takes place).

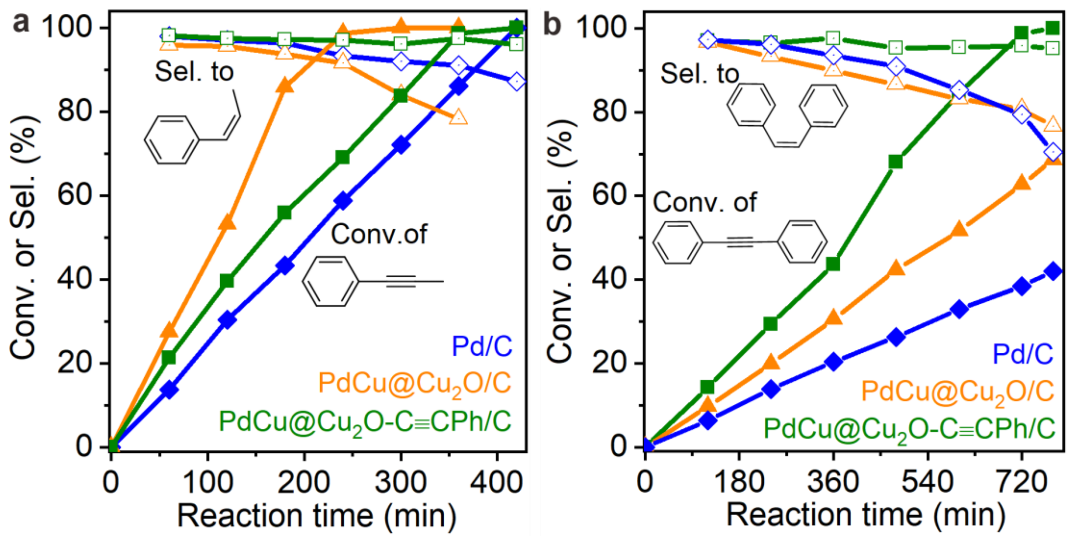

Supplementary Figure 52. Catalytic activity of hydrogenation with phenyl group of internal alkyne with PdCu@Cu_2O-C≡CPh/C and references. (a) 1-Phenyl-1-propyne. (b) Diphenylacetylene. Reaction conditions: 10 mL ethanol; 2 μmol Pd; 1 mmol alkynes (1:500); T = 303 K; pressure = 0.1 MPa H_2 (For PdCu@Cu_2O/C, H_2 was introduced before substrate; for PdCu@Cu_2O-C≡CPh/C, PhC≡CH was introduced before the hydrogenation reaction takes place).

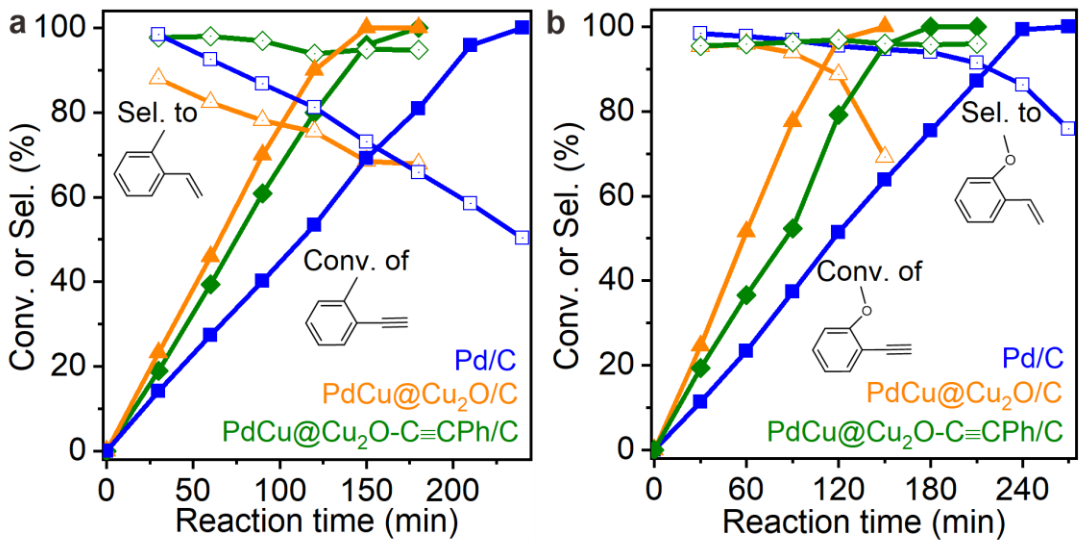

Supplementary Figure 53. Catalytic performance of semi-hydrogenation with a vicinal group of PhC≡CH with PdCu@Cu_2O-C≡CPh/C and references. (a) 2-Ethynyltoluene. (b) 2-Ethynylanisole. Reaction conditions: 10 mL ethanol; 2 μmol Pd; 4 mmol alkynes (1:2,000); T = 303 K; pressure = 0.1 MPa H_2 (For PdCu@Cu_2O/C, H_2 was introduced before substrate; for PdCu@Cu_2O-C≡CPh/C, PhC≡CH was introduced before the hydrogenation reaction takes place).

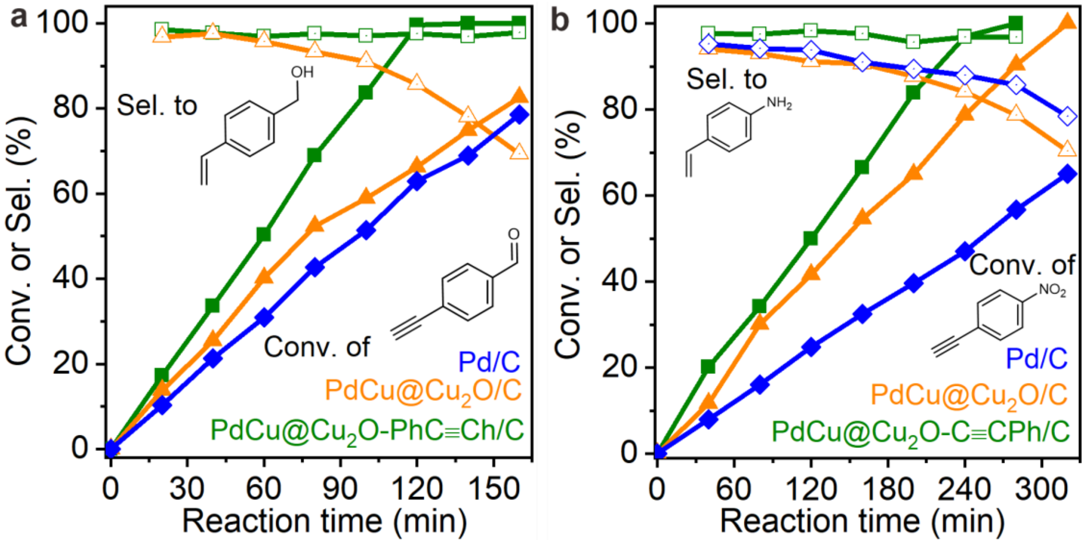

Supplementary Figure 54. Catalytic performance of hydrogenation of organics with C=O groups or –NO_2. (a) 4-Ethynylbenzaldehyde. (b) 4-Nitrophenylacetylene. Reaction conditions: 10 mL ethanol; 2 μmol Pd; 1 mmol organic (1:500); T = 333 K; pressure = 0.1 MPa H_2 (For PdCu@Cu_2O/C, H_2 was introduced before substrate; for PdCu@Cu_2O-C≡CPh/C, PhC≡CH was introduced before the hydrogenation reaction takes place).

Supplementary Table 1. Elemental Analysis of Pd@Cu core-shell nanosheets prepared by using various Cu/Pd ratios.

**Samples**	Initial Cu/Pd ratio	Content (ug/ml) in the samples		Cu/Pd
		Pd	Cu	
Pd@Cu(1:0.2)	1:0.20	10.65	2.02	1:0.19
Pd@Cu(1:0.3)	1:0.30	9.16	2.75	1:0.30
Pd@Cu(1:0.5)	1:0.50	23.60	11.33	1:0.48
Pd@Cu(1:1)	1:1.00	8.38	8.13	1:0.97
Pd@Cu(1:1.25)	1:1.25	6.70	8.30	1:1.24
Pd@Cu(1:1.5)	1:1.50	12.96	19.70	1:1.52
Pd@Cu(1:2)	1:2.00	5.42	10.62	1:1.96
Pd@Cu(1:3)	1:3.00	8.63	25.29	1:2.93

Supplementary Table 2. The XPS analysis of Cu species.

[a] **Catalyst**	[b] K.E./eV		B.E. of	[c] Cu^{1+}/Cu^0
	Cu^{1+}	Cu^0	Cu $2p_{3/2}$	
$PdCu@Cu_2O$	916.0	918.5	932.9	1.69
$PdCu@Cu_2O$-C≡CPh-Hydrogenation 30 ^oC, 2h	916.0	918.5	932.9	1.72

[a] Cu/Pd = 1

[b] Kinetic energy

[c] Intensity ratio of Cu^{1+} to Cu^0 form the analysis of Cu XAES spectra.

Supplementary Table 3. Surface energies ΔE_{surf}(J/m^2) of PdCu models with different ratio of metal Cu and Pd.

Cu/Pd ratio	1/6	3/4	4/3	5/2
a	1.23	0.90	0.59	1.01
b	1.00	0.87	0.61	1.05
c	1.11	0.98	0.43	0.94
d	1.11	0.84	0.32	1.02
e	1.04	0.31	0.54	0.87
f	1.04	0.35	0.71	0.80
g	1.10	0.53	1.01	0.82
h	1.05	0.51	0.50	0.74

Supplementary Table 4. The energies of reactant, intermediates and products along the reaction coordinate as well as the relative barriers of TS1-TS4.

Structures	Reduced PdCu alloy	PdCu @Cu_2O	Clean Pd(111)
PhC≡CH*	-1.67	-0.89	-1.74
TS1	-0.96	-0.22	-0.90
PhC≡CH_2*	-1.72	-2.01	-2.36
TS2	-1.36	-1.35	-1.99
$PhCHCH_2$*	-3.19	-2.65	-3.50
TS3	-2.66	-1.43	-2.58
$PhCHCH_3$*	-2.99	-2.50	-3.65
TS4	-2.61	-2.04	-3.16
$PhCH_2CH_3$	-3.78	-3.72	-3.90
ΔE_a(TS1)	0.71	0.67	0.84
ΔE_a(TS2)	0.36	0.66	0.37
ΔE_a(TS3)	0.53	1.22	0.92
ΔE_a(TS4)	0.38	0.46	0.49

Supplementary References

1. Sheng Y. H. *et al*. The XAFS beamline of SSRF. *Nucl. Sci. Tech.* **26**, 50102-050102 (2015).
2. Ravel, B. & Newville, M. ATHENA, ARTEMIS, HEPHAESTUS: data analysis for X-ray absorption spectroscopy using IFEFFIT. *J. Synchrotron Radiat.* **12**, 537-541 (2005).
3. Funke, H., Chukalina, M. & Scheinost, A.C. A new FEFF-based wavelet for EXAFS data analysis. *J. Synchrotron Radiat.* **14**, 426-432 (2007).
